# *Porphyromonas gingivalis* and *Treponema denticola* Exhibit Metabolic Symbioses

**DOI:** 10.1371/journal.ppat.1003955

**Published:** 2014-03-06

**Authors:** Kheng H. Tan, Christine A. Seers, Stuart G. Dashper, Helen L. Mitchell, James S. Pyke, Vincent Meuric, Nada Slakeski, Steven M. Cleal, Jenny L. Chambers, Malcolm J. McConville, Eric C. Reynolds

**Affiliations:** 1 Oral Health CRC, Melbourne Dental School, Bio21 Institute, The University of Melbourne, Parkville, Victoria, Australia; 2 Department of Biochemistry and Molecular Biology, Bio21 Institute, The University of Melbourne, Parkville, Victoria, Australia; Stanford University, United States of America

## Abstract

*Porphyromonas gingivalis* and *Treponema denticola* are strongly associated with chronic periodontitis. These bacteria have been co-localized in subgingival plaque and demonstrated to exhibit symbiosis in growth *in vitro* and synergistic virulence upon co-infection in animal models of disease. Here we show that during continuous co-culture a *P. gingivalis:T. denticola* cell ratio of 6∶1 was maintained with a respective increase of 54% and 30% in cell numbers when compared with mono-culture. Co-culture caused significant changes in global gene expression in both species with altered expression of 184 *T. denticola* and 134 *P. gingivalis* genes. *P. gingivalis* genes encoding a predicted thiamine biosynthesis pathway were up-regulated whilst genes involved in fatty acid biosynthesis were down-regulated. *T. denticola* genes encoding virulence factors including dentilisin and glycine catabolic pathways were significantly up-regulated during co-culture. Metabolic labeling using ^13^C-glycine showed that *T. denticola* rapidly metabolized this amino acid resulting in the production of acetate and lactate. *P. gingivalis* may be an important source of free glycine for *T. denticola* as mono-cultures of *P. gingivalis* and *T. denticola* were found to produce and consume free glycine, respectively; free glycine production by *P. gingivalis* was stimulated by *T. denticola* conditioned medium and glycine supplementation of *T. denticola* medium increased final cell density 1.7-fold. Collectively these data show *P. gingivalis* and *T. denticola* respond metabolically to the presence of each other with *T. denticola* displaying responses that help explain enhanced virulence of co-infections.

## Author Summary

Unlike the traditional view that most diseases are caused by infection with a single bacterial species, some chronic diseases including periodontitis result from the perturbation of the natural microbiota and the proliferation of a number of opportunistic pathogens. Both *Porphyromonas gingivalis* and *Treponema denticola* have been associated with the progression and severity of chronic periodontitis and have been shown to display synergistic virulence in animal models. However, the underlying mechanisms to these observations are unclear. Here we demonstrate that these two bacteria grow synergistically in continuous co-culture and modify their gene expression. The expression of *T. denticola* genes encoding known virulence factors and enzymes involved in the uptake and metabolism of the amino acid glycine was up-regulated in co-culture. *T. denticola* stimulated the proteolytic *P. gingivalis* to produce free glycine, which *T. denticola* used as a major carbon source. Our study shows *P. gingivalis* and *T. denticola* co-operate metabolically and this helps to explain their synergistic virulence in animal models and their intimate association *in vivo*.

## Introduction

Chronic periodontitis is an inflammatory disease of the supporting tissues of the teeth with a polymicrobial aetiology. However, whilst the concepts of the roles of particular oral bacterial species in disease have changed over the past two decades there is wide consensus that anaerobic, proteolytic, amino acid fermenting species including *Porphyromonas gingivalis*, *Treponema denticola* and *Tannerella forsythia* play a crucial role in initiation and/or progression of disease [1]–[5]. *P. gingivalis* has recently been proposed to be a “keystone pathogen” that through synergistic interactions aids the proliferation of other oral bacterial species resulting in the formation of a pathogenic polymicrobial plaque [3].

We have previously demonstrated in a longitudinal human study that the imminent progression of chronic periodontitis in patients on a maintenance program could be predicted by increases in the relative proportions of *P. gingivalis* and/or *T. denticola* in subgingival plaque above threshold levels [6]. This is consistent with other clinical studies demonstrating that *P. gingivalis* levels in subgingival plaque are predictive of human disease progression [7], [8]. *P. gingivalis* and *T. denticola* are frequently found to co-exist in deep periodontal pockets and have been co-localized to the superfical layers of subgingival plaque as microcolonies adjacent to the pocket epithelium [1], [9]–[13], suggesting possible interbacterial interactions that might contribute towards disease [14], [15]. When co-inoculated intra-orally in animal models of periodontitis *P. gingivalis* and *T. denticola* exhibit a synergistic pathogenesis [16]–[18]. These two bacteria display a symbiotic relationship in nutrient utilization and growth promotion *in vitro* [14], [19] and bimodal coaggregation between *P. gingivalis* and *T. denticola* has been demonstrated [15], [20]–[23], which might explain their co-localization and aid synergistic biofilm production [24]–[26]. *P. gingivalis* and *T. denticola* responded to each other's presence in a polymicrobial biofilm by modulating the abundance of a range of proteins [26]. Together these data suggest there is an intimate relationship between these two species that has evolved to enhance their survival and virulence. However, the physiochemical interactions that result in the observed symbiotic and synergistic effects during *P. gingivalis* and *T. denticola* co-culture remain largely unknown.

In this study we used continuous co-culture to demonstrate that *P. gingivalis* and *T. denticola* symbiotically co-exist and that each bacterium adapts to the presence of the other by modulating gene expression, particularly those genes involved in metabolism and virulence. We show that the presence of *P. gingivalis* caused an up-regulation of *T. denticola* glycine catabolism and that this amino acid supported the growth of *T. denticola*. *T. denticola* conditioned medium induced free glycine production by *P. gingivalis* implying intimate metabolic co-operativity between these species. The up-regulation of *T. denticola* virulence factors in co-culture helps explain the synergistic virulence of *P. gingivalis* and *T. denticola* in animal models of disease.

## Materials and Methods

### Bacterial strains and culture conditions

*P. gingivalis* strain W50 and *T. denticola* ATCC 35405 were obtained from the culture collection of the Oral Health Cooperative Research Centre, The University of Melbourne, and grown in oral bacterial growth medium (OBGM), that meets the growth requirements of both *P. gingivalis* and *T. denticola* [18], [26], [27]. Batch culture was in pre-reduced OBGM at 37°C in a MK3 anaerobic workstation (Don Whitley Scientific, Adelaide, Australia) with a gas composition of 5% CO_2_, 5% H_2_ and 90% N_2_ (BOC Gases, Wetherill Park, Australia). Bacterial cell density was monitored by measuring the absorbance at 650 nm (A_650 nm_).

To initiate the mono-species continuous culture, 300 mL of batch-grown inoculum in exponential growth phase (A_650 nm_ of ∼0.6 for *P. gingivalis* or A_650 nm_ of ∼0.2 for *T. denticola*) was mixed with 600 mL of fresh OBGM and transferred to a Bioflo 110 Modulator Benchtop Fermentor (New Brunswick Scientific, NJ, USA). The culture was maintained at 37°C, continuously agitated at 50 rpm and gassed with a constant stream of anaerobic gas (10% CO_2_ in N_2_) (BOC Gases). After 24 h, medium flow was commenced with OBGM pumped into the fermentor maintaining a working volume of 900 mL, with a flow rate of 39 mL h^−1^, giving a mean generation time of 15.75 h. Samples were collected after the A_650 nm_ of the culture remained stable for a period of ten generations (158 h).

To establish co-culture *P. gingivalis* was inoculated into established *T. denticola* mono-species continuous cultures. After the *T. denticola* samples were harvested from a mono-culture chemostat, co-culture was established by replacing 300 mL of *T. denticola* culture with batch-grown exponential phase *P. gingivalis* (A_650 nm_∼0.6). The medium flow to the fermentor was stopped for 12 h to enable establishment of co-culture, after which medium flow was resumed at 39 mL h^−1^. Three independent biological replicates each of *P. gingivalis* and *T. denticola* mono- and co-cultures were grown.

### Bacterial enumeration

Mono-culture bacterial cell density was determined by correlating A_650 nm_ to a standard curve [28] whilst *P. gingivalis* and *T. denticola* cells numbers in co-culture were determined by quantitative real-time PCR (qPCR) as described previously [6], [26], [28].

### DNA microarray slides

The 60-mer oligonucleotide probes representing predicted open reading frames (ORFs) of the *P. gingivalis* and *T. denticola* genomes to be used for microarray slide preparation were designed using OligoArray 2.l [29] and using the bioinformatic services of Illumina Inc. (CA, USA). The difference in GC content of *P. gingivalis* (48%) and *T. denticola* (38%) genomes was exploited in probe design. BLAST analysis demonstrated that probes from one organism had less than 80% similarity to that from the other organism to minimize the chances of cross-species hybridization. The custom made *T. denticola* ATCC 35405 microarray contained 2518 probes representing 89% of the predicted ORFs in the *T. denticola* genome as described previously in Mitchell *et al.* [30]. The *P. gingivalis* probe set design was based upon the *P. gingivalis* W83 genome sequence [31] and ORF predictions available through The J. Craig Venter Institute (www.jcvi.org), with additional *P. gingivalis* ORFs predicted by the Los Alamos National Laboratory Oralgen project (www.oralgen.lanl.gov). The final set of 1977 probes corresponded to 96% of the predicted *P. gingivalis* ORFs. The full complement of probes for both genomes were printed twice each onto Corning UltraGAPs coated slides using a Virtek Microarray spotter by the Australian Genome Research Facility (Melbourne, Australia). Microarray Sample Pool (MSP) control probes were also included to aid intensity-dependent normalisation [32].

### Total RNA extraction and purification

RNA was isolated using the GenElute Total RNA Purification Kit (Sigma Aldrich, Castle Hill, Australia) and treated with DNase using the Turbo DNA-free kit (Ambion, TX, USA). The amount, integrity and purity of the RNAs were assessed using the Experion Automated Electrophoresis System (Bio-Rad, CA, USA). The absence of genomic DNA contamination from *P. gingivalis* and *T. denticola* in the RNA sample was determined by performing PCR on RNA samples using *TDE0762* (forward: GGCTCCGAATCAAAACGATA, reverse: CTATCGACTCCCCGTTTTCA) and *PG0719* (forward: GCATTGCAGCATAGCGAATA, reverse: GCCGATGGAAAAAGTGTGTT) primer pairs respectively while the respective genomic DNAs were used as positive controls.

### Microarray analysis

Aliquots of *P. gingivalis* and *T. denticola* (100 mL) were harvested from 3 mono-cultures and from 3 co-cultures, with technical replicates harvested 2 days apart. qPCR showed that there were six times as many *P. gingivalis* as *T. denticola* in co-culture (*vide infra*) therefore 6∶1 equivalents of *P. gingivalis*:*T. denticola* mono-culture RNAs were mixed prior to cDNA synthesis (*vide infra*) for use in comparison with the co-culture cDNA. Labeled cDNA hybridization targets were produced using either 5 µg of genomic DNA or 6 µg of purified total RNA template, 5 µg of random hexamers (Life Technologies, MD, USA) and aminoallyl dUTP nucleotides incorporated during cDNA synthesis. The genomic cDNA was produced using Platinum Taq DNA polymerase (Life Technologies) whilst RNA was reverse transcribed using Superscript III Reverse Transcriptase (Life Technologies). cDNA were purified using QlAquick columns (Qiagen, CA, USA) and labeled with monoreactive Cy3 or Cy5 dye (40 nmol) (GE Healthcare Lifesciences, Quebec, Canada). Equal numbers of samples from mono- and co-culture cDNAs were labeled with Cy3 and Cy5 respectively and in reverse combination in order to accommodate for different Cy3 and Cy5 labeling efficiencies. To ensure the specificity of the probes, the dual-genome array was probed with *P. gingivalis* or *T. denticola* labeled cDNA. Five *T. denticola* probes (*TDE0780*, *TDE1033*, *TDE1804*, *TDE2113* and *TDE2213*) were found to cross-hybridize to *P. gingivalis* DNA and six *P. gingivalis* probes (*PG1340*, *PG1473*, *PG1525*, *PG1666*, *PG1731* and *PG2175*) were found to cross-hybridize to *T. denticola* DNA. The expression of these genes was excluded from the subsequent transcriptomic analysis. The microarray hybridization and scanning were conducted essentially as described previously [30], [33] but with 49% formamide in the hybridization buffer and use of 46°C as the hybridization temperature.

### Data analysis

Microarray background subtraction and data analysis were conducted in R statistical environment using GenePix Pro 6.0 and LIMMA as described previously [30], [34]. The Benjamini-Hochberg method was used to control the false discovery rate to correct for multiple testing [35]. Genes with a fold change equal to or above 1.4 and an adjusted *p*≤0.05 were considered to be significantly differentially regulated. Annotation and putative protein functions were based on the National Center for Biotechnology Information Refseq database and Clusters of Orthologous Groups of protein (COG) database [36]. Operon predictions, COG assignments and COG functional categories were obtained from the Microbes Online database [37]. Microarray data are available in the ArrayExpress database (www.ebi.ac.uk/arrayexpress) under accession number E-MTAB-2214 (*P. gingivalis*) and E-MTAB-2257 (*T. denticola*).

### Validation of microarray results using quantitative reverse transcription PCR (qRT-PCR)

Differential expression of selected genes was validated by qRT-PCR of cDNAs using SYBR Green-based detection on a Rotor Gene 3000 system (Corbett Research, Sydney, Australia) as reported previously [30]. To normalize the amount of mRNA in each reaction, a pool of six reference genes *TDE2535*, *TDE0872*, *TDE1208*, *TDE1999*, *TDE1226* and *TDE0002* [30] was assessed for expression stability using the geNorm program [38]. Analysis indicated that the geometric mean of the three most stable genes *TDE2535*, *TDE1208* and *TDE0872* with average pair-wise variations of 0.146 was sufficient to calculate a normalization factor for each sample. Primers for the validation gene set *TDE1624*, *TDE1625*, *TDE1626*, *TDE1627*, *TDE1259*, *TDE2119*, *TDE2120*, *TDE0405*, *TDE0762*, *TDE1669*, *TDE0387*, *TDE0627* and *TDE0832* were designed using Primer3 [39] (Table S1).

### Scanning electron microscopy

Co-cultures from continuous culture were collected then processed and imaged using a Philips XL30 field-emission scanning electron microscope at a voltage of 2 kV as described previously [30].

### Analysis of glycine concentration

Batch cultures of *P. gingivalis* (50 mL) with a starting cell density of 1×10^7^ cells were grown using a) OBGM, b) OBGM supplemented 1∶1 with phosphate buffered saline (PBS) (OBGM/PBS) and c) OBGM supplemented 1∶1 with cell-free *T. denticola* conditioned growth medium (OBGM/*T. denticola* conditioned medium). *T. denticola* conditioned medium was prepared by filtering (0.1 µm) a 7-day culture of *T. denticola* grown in OBGM. Aliquots of the culture medium (30 µL) were collected and immediately diluted with 70 µL of deionized water, 300 µL of CHCl_3_ and 100 µL of CH_3_OH. The extracted medium was snap-frozen on liquid nitrogen and stored at −80°C for later gas chromatography–mass spectrometry (GC-MS) analysis [40] (Protocol S1). A glycine standard curve was obtained by the addition of various amounts of glycine to *T. denticola* conditioned medium (which had no detectable free glycine) in preference to use of pure glycine standards to account for matrix effects from the medium. The difference in free glycine levels relative to that at t = 0 h for each replicate was expressed as a function of *P. gingivalis* cell numbers in different media. A regression line was fitted using a linear mixed modelling approach, which allowed the regression lines to be fitted with random slopes by considering the fixed effects for the different growth medium and time interaction (SPSS version 20, IBM, IL, USA). For the determination of the total glycine content of cell-free media samples were freeze-dried and hydrolyzed at 150°C for 2 h in the presence of 6 M HCl containing phenol (1% v/v).

### Isotope-labeled glycine studies

[U-^13^C]glycine (Cambridge Isotope Laboratories, MA, USA) was added to a final concentration of 5 mM to a 24 h *T. denticola* culture in OBGM. Bacterial culture (1.5 mL) was collected every 24 h for 8 days and passed through a 0.22 µM filter to obtain a cell-free fraction. Samples were kept at −80°C until further analysis. Sample preparation for ^13^C-nuclear magnetic resonance (NMR) spectroscopy and spectra analyses were conducted by Metabolomics Australia (Melbourne, Australia) as previously published [40].

### Thiamine pyrophosphate (TPP) production by *P. gingivalis*

*Escherichia coli* JRG902 [41] and *E. coli* JW3957-1, a TPP auxotrophic strain (*ΔthiE764::kan*), were obtained from the Keio collection [42]. *E. coli* strains were grown in M9 medium [43] with different combinations of TPP (5.88 nM) and/or cell-free *P. gingivalis* conditioned medium that was obtained by growing *P. gingivalis* in OMIZ-M/TD [44] without TPP and filtering (0.2 µM) the culture. Growth was measured by A_650 nm_.

## Results

### *P. gingivalis* and *T. denticola* continuous culture

**Figure 1:**
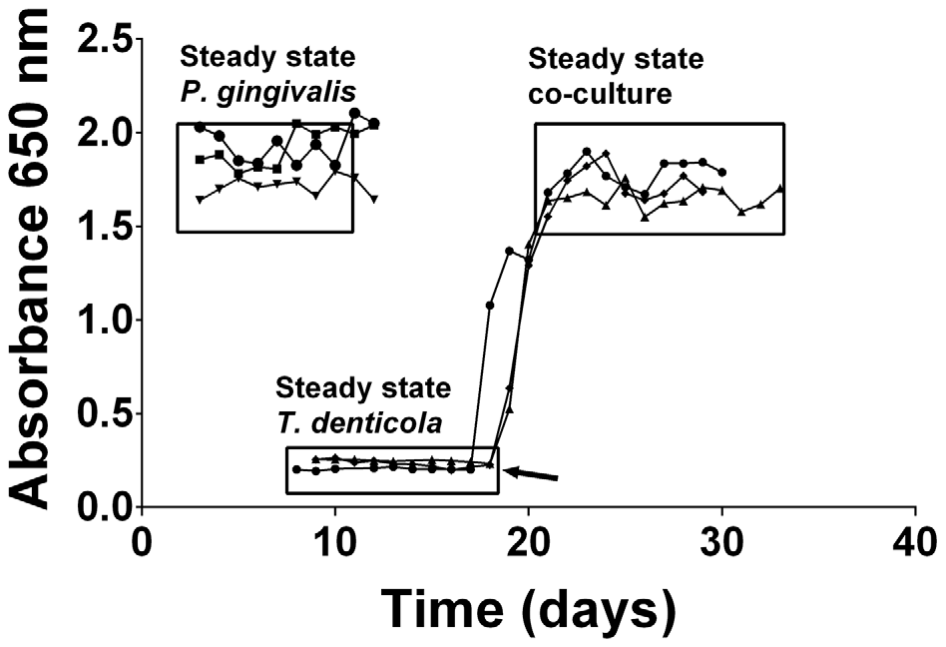
Continuous culture of *P. gingivalis* and *T. denticola* mono- and co-cultures. Cell density of *P. gingivalis* and *T. denticola* mono- and co-cultures from three independent continuous cultures in OBGM with the dilution rate of 0.044 h^−1^ and mean generation time of 15.8 h as determined by measuring A_650 nm_. The arrow shows the addition of *P. gingivalis* to a steady state *T. denticola* culture.

**Figure 2:**
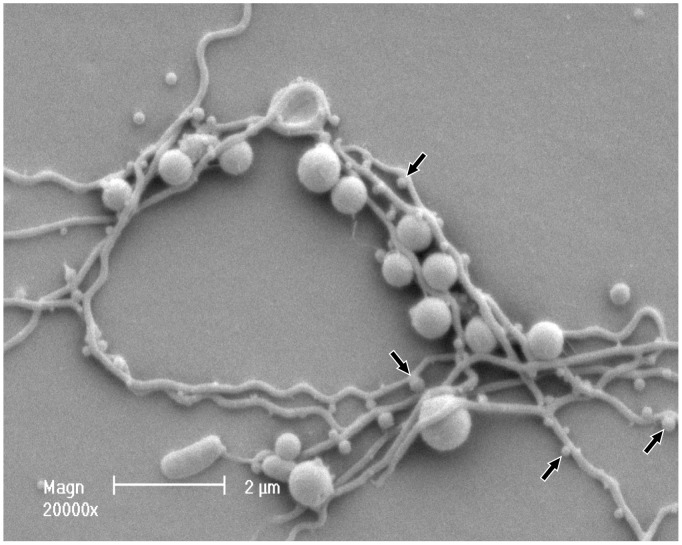
Scanning electron micrograph of *P. gingivalis* and *T. denticola* grown in continuous culture. Co-culture was collected, fixed on a coverslip, dehydrated, covered with colloidal silver, gold-coated and imaged using a Philips XL30 field-emission scanning electron microscope. Electron micrographs showed that *P. gingivalis* and *T. denticola* coaggregated. *T. denticola* is a long helical shaped spirochete with an average length of 5 to 20 µm. *P. gingivalis* is a coccobacillus with an average diameter of 1 µm. Putative *T. denticola* outer sheath vesicles and/or *P. gingivalis* outer membrane vesicles are indicated by arrows along the length of *T. denticola*.

Mono- and co-cultures of *P. gingivalis* and *T. denticola* were established in continuous culture using OBGM. Three independent continuous mono-cultures of *P. gingivalis* entered steady state 3 days after establishment with an average absorbance (A_650 nm_) of 1.86±0.14 equating to 3.9±0.3×10^9^ cells mL^−1^. The three *T. denticola* mono-cultures also reached steady state approximately 8 days after inoculation with an average A_650 nm_ of 0.23±0.02 equating to 7.7±0.7×10^8^ cells mL^−1^ (Figure 1). Co-cultures of *P. gingivalis* and *T. denticola* entered steady-state after 4 days with an average A_650 nm_ of 1.73±0.09 (Figure 1). There were 6.0±0.7×10^9^ *P. gingivalis* cells mL^−1^ and 1.0±0.1×10^9^ *T. denticola* cells mL^−1^ under steady state conditions for the three biological replicates as determined by qPCR. Thus cell densities of both *P. gingivalis* and *T. denticola* increased significantly, by 54% and 30% (*p*<0.01) respectively, in co-culture compared with mono-culture. Interestingly, the 50% increase in total bacterial cell number during co-culture was not reflected by a concomitant increase in absorbance. SEM analysis of a co-culture showed that there was considerable coaggregation between the species with *P. gingivalis* adhering along the entire length of *T. denticola* cells, which would explain the lower culture A_650 nm_ than would be expected for this density of discrete cells (Figure 2).

### Differential gene expression in co-culture

The expression of 184 *T. denticola* genes (6.6% of the genome) and 134 *P. gingivalis* genes (7.4% of the genome) was differentially regulated (adjusted *p*≤0.05 and fold change of ≥1.4) in co-culture in comparison with mono-culture. Many of these genes were predicted to be polycistronic, with 33 predicted *T. denticola* and 16 predicted *P. gingivalis* operons containing two or more differentially co-regulated genes. The direction of change of expression was consistent within the predicted operons, with no single operon containing both up-regulated and down-regulated genes (Table S2; Table S3). Differentially expressed genes were sorted into functional categories on the basis of clusters of orthologous groups (COG) [45]. The majority of *T. denticola* (103/184) and *P. gingivalis* (80/134) differentially expressed genes encoded proteins that belong to category R (general function prediction), category S (function unknown) or the not assigned category (Table 1).

**Table 1:** *T. denticola* and *P. gingivalis* genes differentially expressed during continuous co-culture relative to mono-culture, grouped by COG category.

	*	No. of genes in *P. gingivalis*	*Differentially expressed P. gingivalis genes*		No. of genes in *T. denticola*	*Differentially expressed T. denticola genes*	
	COG		TOTAL	% of COG		TOTAL	% of COG
Information storage and processing	J	117	6	5.1	138	1	0.7
	K	38	2	5.3	62	8	12.9
	L	88	10	11.4	86	2	2.3
Cellular processes and signaling	D	15	0	0.0	15	0	0.0
	V	27	3	11.1	93	5	5.4
	T	27	4	14.8	85	9	10.6
	M	109	2	1.8	101	9	8.9
	N	6	1	16.7	27	0	0.0
	U	15	1	6.7	35	3	8.6
	O	52	7	13.5	55	10	18.2
Metabolism	C	73	1	1.4	61	3	4.9
	G	48	1	2.1	73	4	5.5
	E	73	1	1.4	100	15	15.0
	F	52	1	1.9	45	2	4.4
	H	89	7	7.9	51	3	5.9
	I	33	4	12.1	40	3	7.5
	P	48	3	6.3	104	4	3.8
	Q	7	0	0.0	5	0	0.0
Poorly or not characterized	R	128	8	6.3	188	10	5.3
	S	62	2	3.2	159	13	8.2
	N/A	710	70	9.9	1263	80	6.3
	Total	1817	134	7.4	2786	184	6.6

* One-letter abbreviations for the functional COG categories: J, translation, ribosomal structure and biogenesis; K, transcription; L, replication, recombination and repair; D, cell cycle control, cell division, chromosome partitioning; V, defense mechanisms; T, signal transduction mechanisms; M, cell wall/membrane/envelope biogenesis; N, cell motility; U, intracellular trafficking, secretion, and vesicular transport; O, posttranslational modification, protein turnover, chaperones; C, energy production and conversion; G, carbohydrate transport and metabolism; E, amino acid transport and metabolism; F, nucleotide transport and metabolism; H, coenzyme transport and metabolism; I, lipid transport and metabolism; P, inorganic ion transport and metabolism; Q, secondary metabolites biosynthesis, transport and catabolism; R, general function prediction only; S, function unknown.

### *T. denticola* differentially expressed genes

Thirty-four differentially expressed *T. denticola* genes were assigned to COGs related to metabolism, with 15 of these clustering in category E (amino acid transport and metabolism), representing 15% of the total genes assigned to this COG (Table 1). *TDE0392* and *TDE0389* which encode the putative Fe-S-dependent β subunits (HgdCA, HgdB) of (R)-2-hydroxyglutaryl-CoA dehydratase, an iron-sulfur cluster (4Fe-4S)-dependent enzyme involved in the fermentation of glutamate, as well as *TDE0387* which encodes its 4Fe-4S-dependent activator HgdC, were down-regulated during co-culture. In addition, the gene encoding carbamate kinase (*TDE2476*) was down-regulated suggesting reduced glutamate catabolism by *T. denticola* when co-cultured with *P. gingivalis*. In contrast genes encoding enzymes involved in the glycine cleavage system (GcvP1, GcvP2 and GcvH), the glycine reductase system (Protein B2) and oligopeptide/dipeptide/amino acid transporters (OppA, TDE1067, TDE0985) were up-regulated suggesting an increased glycine catabolism by *T. denticola*.

The increased expression of genes encoding components of the glycine cleavage and glycine reductase systems was confirmed by qRT-PCR using *TDE2535*, *TDE0872* and *TDE1208* as stable reference genes. There was a significant correlation between the expression ratios determined by both microarray and qRT-PCR (R^2^ = 0.9839) (Figure S1). This comparison revealed a slight compression of the gene expression data from the DNA microarray analysis and gave a higher up-regulation of components of the glycine cleavage and glycine reductase systems with a >1.4 fold up-regulation of TDE1627, the T-protein of the glycine cleavage system and *TDE2120*, the GrdE2 of the glycine reductase system (Table 2).

**Table 2:** Expression of *T. denticola* genes encoding enzymes involved in glycine or glycine-related metabolism during co-culture with *P. gingivalis*.

Protein function(s)	Gene		Expression fold change (co-culture versus mono-culture)	
			Microarray	qRT-PCR
Glycine cleavage	*TDE1624* P-protein subunit 2		1.7^1^	1.8^1^
	*TDE1625* P-protein subunit 1		1.5^1^	1.6^1^
	*TDE1626* H-protein		1.4^1^	1.7^1^
	*TDE1627* T-protein		1.3^1^	1.8^1^
	*TDE1629* L-protein		1.3^1^	ND
Conversion of methylene THF to 10-formylTHF	*TDE0013* MethyleneTHF dehydrogenase/MethenylTHF cyclohydrolase		1.5^1^	ND
Conversion of 10-formylTHF to formate	*TDE0019* FormylTHF synthase		1.0	ND
Glycine reductase system	Protein B1	*TDE0078* GrdB1	−1.3^1^	ND
		*TDE0077* GrdE1	−1.2^1^	ND
	Protein B2	*TDE2119* GrdB2	1.4^1^	2.1^1^
		*TDE2120* GrdE2	1.3^1^	1.7^1^
	Protein A	*TDE0745* GrdA	−1.1	ND^2^
	Protein C	*TDE0240* GrdC	−1.1	ND
		*TDE0239* GrdD	−1.1	ND
Conversion of acetyl-phosphate to acetate	*TDE0933* Acetate kinase		1.2^1^	ND
Alanine/Glycine cation symporter (AGCS)	*TDE1259* Na+/Alanine-glycine symporter		1.4^1^	1.8^1^

1 represents fold change with adjusted *p*<0.05.

2 Not determined.

The COG category with the most differentially expressed genes was O (posttranslational modification, protein turnover, chaperones), where 10 genes (18.2%) had altered expression, nine of which were down-regulated (Table 1). Notably, genes encoding virulence factors were amongst the most up-regulated during co-culture, including those encoding the major sheath protein (TDE0405), the dentilisin protease complex (TDE0761 and TDE0762) and cystalysin (TDE1669). Altered chemotactic responses were also evident with reduction in expression of five receptors, the methyl-accepting chemotaxis proteins TDE0338, TDE0484, TDE1009, TDE2270 and TDE2496. Also down-regulated were genes that encode a FeS assembly ATPase (SufC), FeS assembly protein (SufB) and peptidyl-prolyl cis-trans isomerases (TDE1925, TDE2287, TDE2391).

### *P. gingivalis* differentially expressed genes

Co-culture with *T. denticola* had no effect on the transcription of *P. gingivalis* genes belonging to categories Q (secondary metabolites biosynthesis, transport and catabolism) and D (cell cycle control, cell division, chromosome partitioning) (Table 1). Furthermore, little effect on the transcription of genes in categories M (cell wall/membrane/envelope biogenesis), C (energy production and conversion), G (carbohydrate transport and metabolism), E (amino acid transport and metabolism) and F (nucleotide transport and metabolism) was observed. Six differentially expressed genes in category H (coenzyme transport and metabolism) were up-regulated, four of which (*thiH*, *thiG*, *thiE/D*, *thiS*) occur in a predicted five gene operon (*PG2107-11*) suggesting increased thiamine biosynthesis during co-culture. In addition *PG2010* (*thiC*) was also significantly up-regulated (adjusted *p* = 0.01) but did not meet the 1.4-fold cut-off criterion. The putative thiamine transporter, PnuT (*PG1898*) was significantly up-regulated 1.5 fold (Table S3). Three genes involved in the initial stages of fatty acid biosynthesis *fabG*, *fabF*, *acpP* were down-regulated (Table S3).

### Free glycine use by *T. denticola*

**Figure 3:**
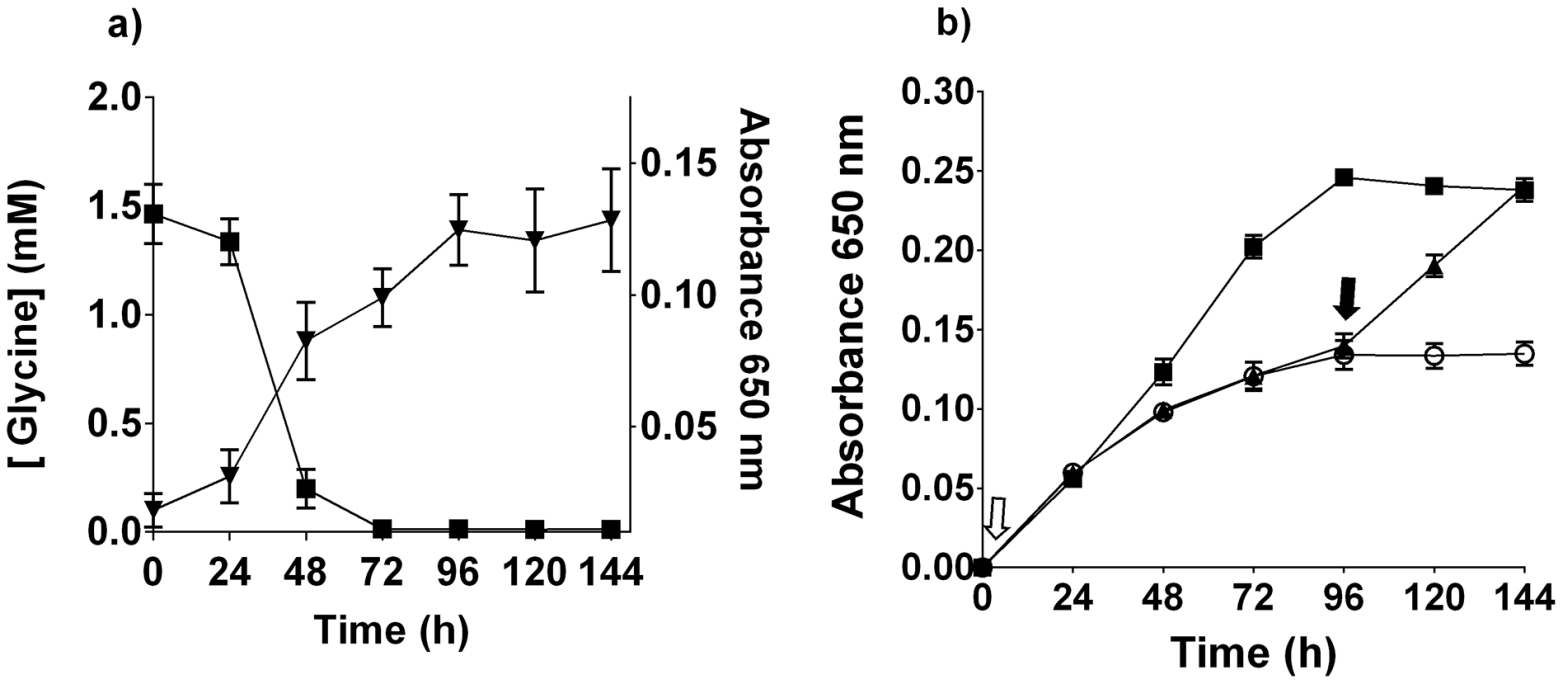
*T. denticola* growth and glycine. (a) The concentration of free glycine in *T. denticola* culture (black square; left axis). *T. denticola* growth curve in the same medium (black inverted triangle; right axis). Data points are the mean and standard deviation of three biological replicates. (b) Glycine (10 mM) was added to OBGM either before inoculation with *T. denticola* (black square, open arrow) or at 96 h after inoculation (black triangle, filled arrow) and bacterial growth was determined by A_650 nm_ measurement. *T. denticola* culture with no added glycine (white circle). Results are expressed as mean ± standard deviation obtained from eight replicates.

The increased transcription of genes encoding enzymes in glycine metabolism in *T. denticola* when co-cultured with *P. gingivalis* prompted us to investigate *T. denticola* glycine metabolism further. Suspension of *T. denticola* in OBGM containing a starting concentration of 1.46 mM glycine, resulted in the rapid depletion of this amino acid over 72 h, coinciding with entry into stationary growth (Figure 3a). Further supplementation of the OBGM to 10 mM glycine enhanced *T. denticola* growth, resulting in a 1.75-fold increase in final cell density. Addition of glycine to a stationary *T. denticola* culture caused the resumption of bacterial growth (Figure 3b). These data show that *T. denticola* consumed free glycine and that glycine availability had a significant impact on *T. denticola* growth.

### Metabolic fate of free glycine

**Figure 4:**
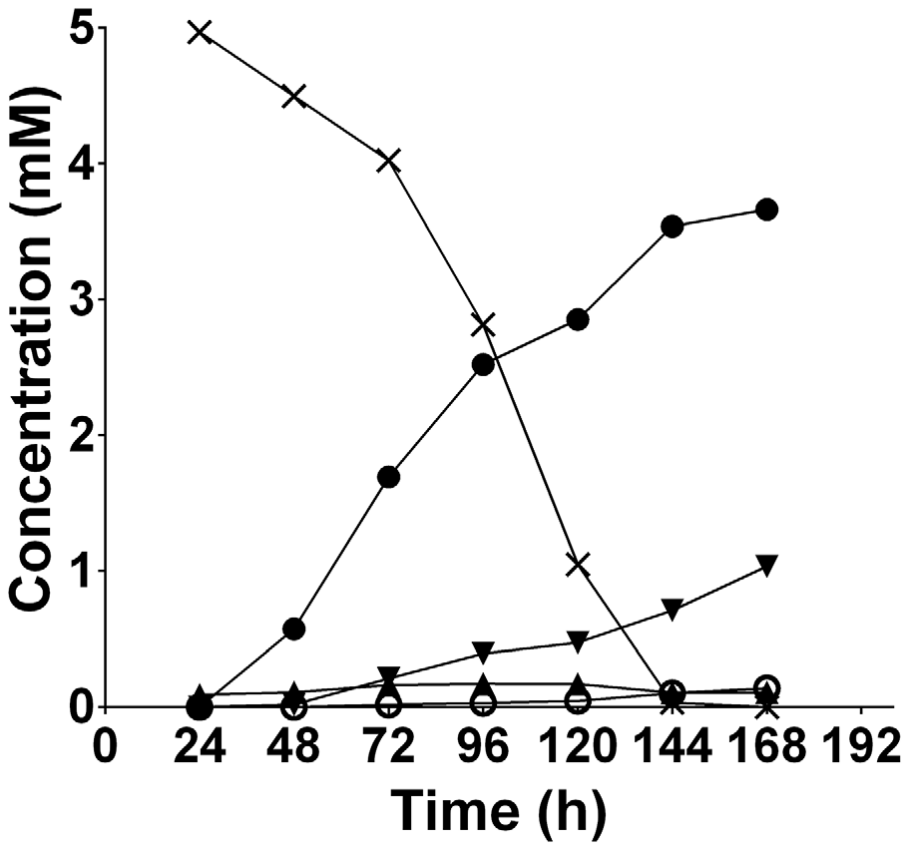
The extracellular products of *T. denticola* [U-^13^C]glycine fermentation. [U-^13^C]glycine (5.00 mM) was added to a 24 h *T. denticola* culture and aliquots were collected every 24 h, filtered and the identity and the quantity of the ^13^C-labeled compounds was determined using NMR spectroscopy. black cross, [U-^13^C]glycine; black circle, [U-^13^C]acetate; black inverted triangle, dual or uniformly-labeled lactate; black triangle, [U-^13^C]bicarbonate; white circle, dual or uniformly-labeled alanine.

Direct evidence for glycine catabolism in *T. denticola* was provided by metabolic labeling with 5 mM [U-^13^C]glycine. [U-^13^C]glycine was added to a 24 h batch-grown *T. denticola* culture and consumption of ^13^C-glycine and production of ^13^C-labeled end-products determined by ^13^C-NMR analysis of the culture medium (Figure S2). [U-^13^C]glycine was completely consumed by 144 h, with production of ^13^C-acetate (3.67 mM) and ^13^C-lactate (1 mM) (Figure 4). The major isotopomers of acetate and lactate were uniformly labeled, although low levels of [1-^13^C]acetate and/or [2-^13^C]acetate, were also detected (data not shown).

Catabolism of ^13^C-glycine was also associated with the production of H^13^CO_3_. The yields of H^13^CO_3_ (0.17 mM) are likely to be an underestimate due to equilibration with the CO_2_ enriched atmosphere above the medium. A small amount of ^13^C-labeled alanine was also produced with ∼0.14 mM being detected at the 168 h time point.

### *T. denticola* conditioned medium stimulates free glycine production by *P. gingivalis*

**Figure 5:**
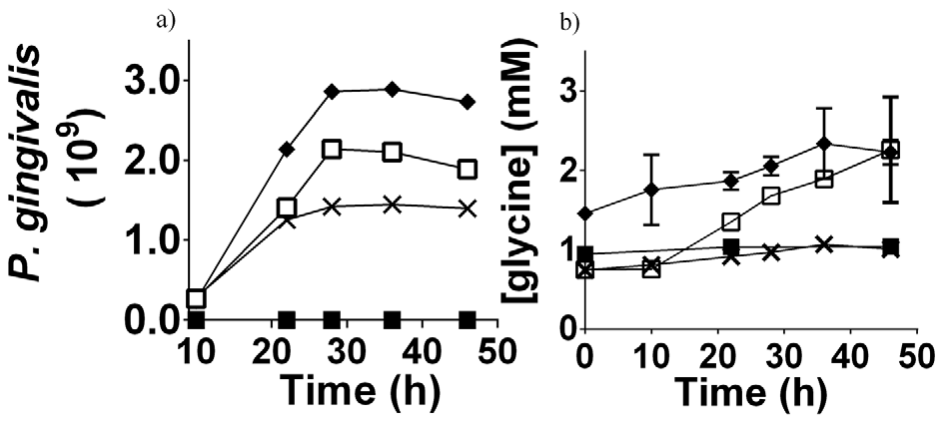
*P. gingivalis* cell numbers and free glycine content in different cultures. a) The cell numbers of *P. gingivalis* in different media as determined by absorbance at 650 nm. b) The concentration of free glycine in different *P. gingivalis* cultures over time, as determined by GC-MS. Data shown are the average of three biological replicates. *P. gingivalis* grown in:- OBGM – black diamond; OBGM/PBS – black cross; OBGM/*T. denticola* conditioned medium – white square. Uninoculated OBGM/*T. denticola* conditioned medium – black square.

**Figure 6:**
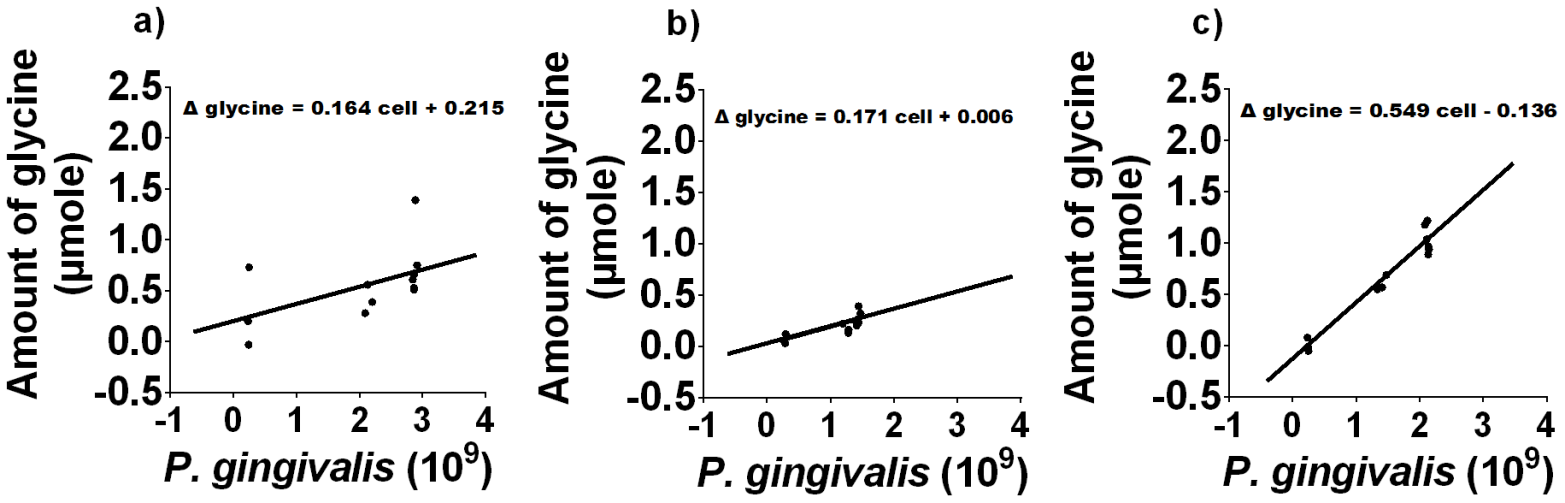
Free glycine production during *P. gingivalis* growth. The difference in the amount of free glycine relative to that at t = 0 h as a function of *P. gingivalis* cell numbers in a) OBGM/PBS, b) OBGM and c) OBGM/*T. denticola* conditioned medium. A regression line was fitted using a linear mixed modelling approach. The slope represents the amount of glycine produced/10^9^ *P. gingivalis* cells.

Increased expression of *T. denticola* genes encoding glycine catabolic pathways during co-culture suggested that there may be increased glycine availability. We therefore determined if *P. gingivalis* growth could provide this additional glycine and if *T. denticola* could stimulate *P. gingivalis* glycine production. When *P. gingivalis* was grown in OBGM there was a small increase in free glycine (Figure 5) while no increase in free glycine was evident in uninoculated OBGM over the same time period (data not shown). When *P. gingivalis* was grown in OBGM/*T. denticola* conditioned medium free glycine increased from 0.75±0.04 mM at time 0 h to 2.26±0.66 mM after 46 h, a significant difference of 1.51 mM (*p*<0.01) whereas the free glycine in the OBGM/PBS control culture increased only 0.26±0.04 mM (*p*<0.01) (Figure 5) after 46 h. In contrast free glycine content was unchanged in the uninoculated OBGM/*T. denticola* conditioned medium control (Figure 5). To account for the differing number of *P. gingivalis* cells in the different media over time and the influence of this on free glycine generation, the change in glycine concentration was expressed as a function of *P. gingivalis* cell number. A regression line was fitted using a linear mixed modelling approach with the slope representing glycine production per 10^9^ *P. gingivalis* cells (Figure 6). Glycine production by *P. gingivalis* grown in OBGM/PBS was 0.171±0.012 µmole glycine/10^9^ cells which was not statistically different from that determined in OBGM at 0.164±0.020 µmole glycine/10^9^ cells (*p*>0.10) (Figure 6). However, free glycine production by *P. gingivalis* in OBGM/*T. denticola* conditioned medium was 0.549±0.090 µmole/10^9^ cells, which was more than three times that observed in OBGM or OBGM/PBS (*p*<0.01).

To determine the source of this increased free glycine the free and total glycine concentrations were determined prior to and 48 h after *P. gingivalis* inoculation in OBGM/*T. denticola* conditioned and OBGM/PBS media. Peptide-bound glycine (total - free) decreased by 1.264±0.143 µmole/10^9^ cells in the OBGM/*T. denticola* conditioned medium, significantly (*p*<0.01) more than the 0.733±0.056 µmole/10^9^ cells decrease in OBGM/PBS. These data indicate a higher rate of peptide hydrolysis and release of free glycine by *P. gingivalis* in the OBGM/*T. denticola* conditioned medium. Total glycine decreased by 0.765±0.090 µmole/10^9^ cells in the OBGM/*T. denticola* conditioned medium, compared with the 0.583±0.030 µmole/10^9^ cells decrease in OBGM/PBS, indicating a slightly higher rate of uptake by *P. gingivalis* in the OBGM/*T. denticola* conditioned medium.

### *P. gingivalis* TPP production

**Figure 7:**
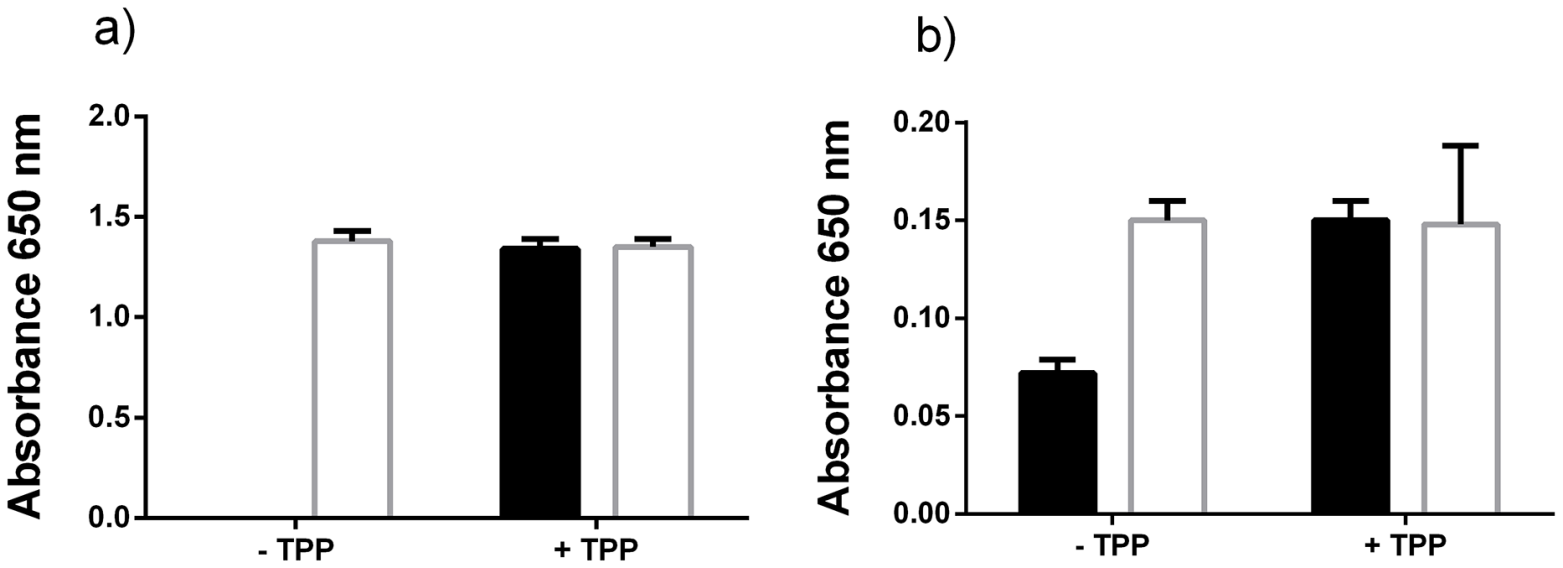
Excess thiamine pyrophosphate production by *P. gingivalis*. The *E. coli* TPP auxotrophic strain JW3957-1 (black shading) and the parent strain JRG902 (white shading) were cultured in M9 growth medium (that lacks thiamine) supplemented with either (a) uninoculated *P. gingivalis* medium (that lacks thiamine) or (b) cell-free *P. gingivalis* spent medium. The bacterium was cultured with or without TPP addition (5.88 nM).

The increased transcription of *P. gingivalis* genes during co-culture that encoded thiamine biosynthesis and transport-related proteins prompted us to examine whether *P. gingivalis* produces excess thiamine that could be used by *T. denticola*, a thiamine auxotroph. Thiamine pyrophosphate (TPP) is a micronutrient that is required in extremely low concentrations for bacterial growth that are difficult to detect biochemically. We therefore used an *E. coli* auxotrophic strain, JW3957-1 to determine excess thiamine production and release by *P. gingivalis*. *E. coli* JW3957-1 was unable to grow in M9 minimal medium supplemented with uninoculated *P. gingivalis* growth medium unless it was supplemented with 5.88 nM TPP. In contrast the *E. coli* parent strain JRG902 was able to grow to a similar cell density with or without 5.88 nM TPP (Figure 7a). The addition of *P. gingivalis* cell-free spent medium caused inhibition of *E. coli* JRG902 growth by an undefined mechanism that was independent of TPP addition (Figure 7b). However the addition of *P. gingivalis* cell-free spent medium did enable limited growth of *E. coli* JW3957-1, indicating that there was some available TPP in the medium that had been produced and released by *P. gingivalis* (Figure 7b). Addition of 5.88 nM TPP resulted in a similar final cell density of both *E. coli* JW3957-1 and JRG902.

## Discussion

*P. gingivalis* and *T. denticola* are frequently found together in subgingival plaque samples taken from diseased periodontal sites [1], [6], [10] as spatially co-localized micro-colonies on the surface of the plaque adjacent to the host pocket epithelium [12], [13]. This implies a strong ecological relationship and potential interactions that may contribute to the progression of chronic periodontitis. In this study, we showed that the sum of the interactions (including competition and cooperation) between *P. gingivalis* and *T. denticola* resulted in sustained interspecies growth symbiosis in continuous culture over an extended time frame. In addition, the two species reached a reproducible steady state with a cell density significantly higher than in mono-culture that strongly suggests metabolic synergy.

A dual species transcriptome analysis determined that *T. denticola* and *P. gingivalis* responded to each other in co-culture by altering the expression of a substantial proportion of genes, notably those involved in *T. denticola* metabolism and virulence. The shift in metabolism was evidenced by altered expression in genes encoding proteins involved in iron acquisition, utilization and storage by each species, altered glutamate and glycine catabolism by *T. denticola* and changes in *P. gingivalis* fatty acid and thiamine pyrophosphate synthesis. This suggests that there may be metabolites produced by each species that enables the other to bypass or alter specific metabolic processes. The up-regulation of the expression of *P. gingivalis* genes encoding thiamine pyrophosphate biosynthesis indicated an increased production of TPP by *P. gingivalis*. This may be a result of competition with *T. denticola*, which is auxotrophic for TPP, or may indicate that that there is some cross-feeding of TPP from *P. gingivalis* to *T. denticola*. Due to the difficulties of biochemically measuring nanomolar concentrations of TPP we used an *E. coli* TPP auxotrophic strain to demonstrate that *P. gingivalis* produces free TPP that can be utilized by other bacterial species. This indicates that there is a likelihood that *T. denticola* benefits from *P. gingivalis* TPP biosynthesis and release in co-culture.

Genes encoding proteins involved in iron storage, heme acquisition and thioredoxin were down-regulated in *P. gingivalis* co-cultured with *T. denticola* indicating that *P. gingivalis* experienced a metabolic shift as a result of co-culture. *P. gingivalis* is auxotrophic for porphyrin, therefore also for heme and cobalamins as it lacks key enzymes in the early steps of porphyrin biosynthesis [31]. The *P. gingivalis* gene *hmuY* (*PG1551*) that encodes an outer-membrane hemin binding protein important in heme acquisition was the most down-regulated *P. gingivalis* gene during co-culture with *T. denticola*, supporting our recent finding of a significant decrease in HmuY abundance in a polymicrobial biofilm containing *P. gingivalis* and *T. denticola* [26]. Succinate produced by *T. denticola* has been reported to alleviate the *P. gingivalis* requirement for heme during growth in heme-limited medium [14], [46].

In addition fatty acid cross-feeding has previously been demonstrated between *P. gingivalis and T. denticola* [15] which is consistent with the down-regulation of genes encoding enzymes participating in the initial stage of fatty acid synthesis found in our study. Hence, these results suggest that the presence of *T. denticola* helps *P. gingivalis* reduce energy consuming processes which may explain the increase in cell biomass of *P. gingivalis* grown in the presence of *T. denticola*.

The coaggregation of *T. denticola* with *P. gingivalis* in co-culture as shown by SEM would assist *T. denticola* in uptake of nutrients produced by *P. gingivalis*, and *vice versa*. In the polymicrobial biota of subgingival dental plaque, which is subject to the flow of gingival crevicular fluid, the ability of *T. denticola* to adhere to other bacteria and to stimulate production of metabolites such as glycine by other species to provide energy and to compensate for it auxotrophies [44], [47] would be of significant benefit to survival and thus virulence. The motility of *T. denticola* and chemotactic responses would also be significant to its survival. Decreased expression of genes encoding MCP chemotaxis receptors by *T. denticola* in co-culture with *P. gingivalis* relative to mono-culture indicates that several substrates, possibly glycine and TPP are in greater abundance in the co-culture such that chemotaxis to a more prefered environment is of lesser importance.

**Figure 8:**
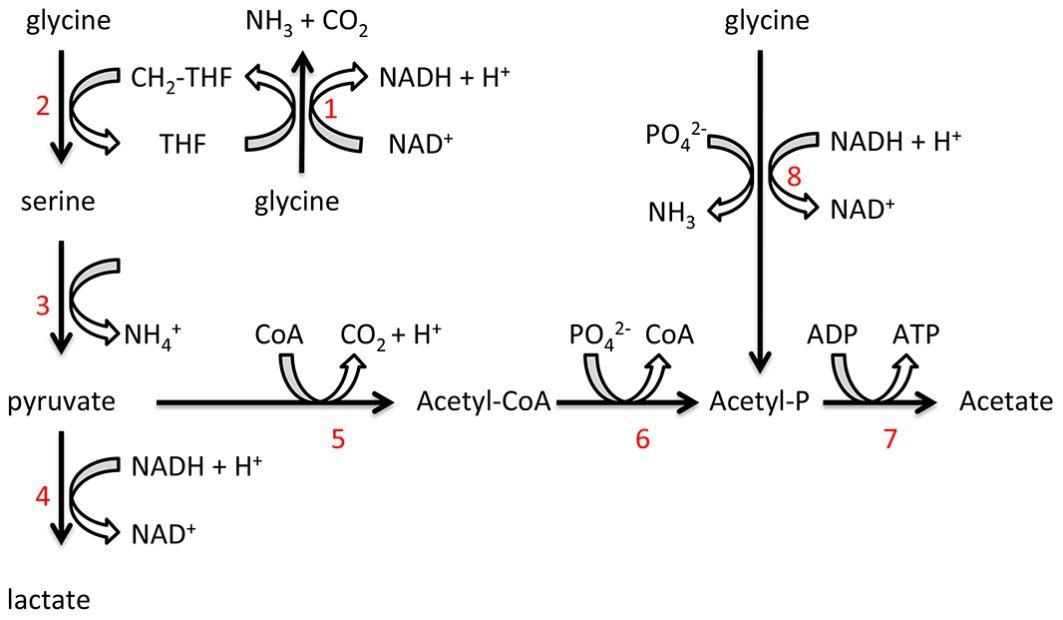
Proposed *T. denticola* glycine catabolic pathways. Glycine can be oxidized by the glycine cleavage system (1), producing NH_3_, CO_2_ and CH_2_-THF. Glycine and CH_2_-THF can be condensed to form serine by serine hydroxymethyltransferase (2). Serine is deaminated to produce pyruvate by serine dehydratase (3). Lactate dehydrogenase (4) catalyzes the interconversion of pyruvate and lactate with concomitant interconversion of NADH and NAD^+^. Pyruvate can also be metabolized to acetate by pyruvate-ferredoxin oxidoreductase (5), phosphate acetyltransferase (6) and acetate kinase (7). Glycine can also be reduced to acetyl-P by the glycine reductase system (8).

Genes involved in *T. denticola* glutamate metabolism were down-regulated in co-culture. This potential shift in catabolism may be related to reduced FeS-cofactor biosynthesis and competition as *P. gingivalis* has a preference for glutamate and aspartate [48], [49]. Significant up-regulation in the expression of *T. denticola* genes encoding peptidases and enzymes involved in glycine catabolism in co-culture relative to mono-culture was also observed. *T. denticola* is predicted to have an alanine/glycine cation symporter (TDE1259), a complete glycine cleavage system and the glycine reductase system [47]. Glycine is utilized as an important energy and carbon source in many proteolytic clostridia and Gram-positive bacteria mainly via the activity of the glycine reductase system [50]. We have previously identified, using mass spectrometry, some enzymes of these pathways in *T. denticola* which were abundant suggesting glycine catabolism may be a major energy source for this spirochete [27]. The importance of the glycine reductase system is also suggested by the recent report demonstrating inhibition of *T. denticola* growth through the impairment of selenoprotein production such as Protein A and B of the glycine reductase system by stannous salts and auranofin [51]. In support of the contention that glycine catabolism is important in *T. denticola* free glycine was rapidly depleted from *T. denticola* growth medium and the addition of glycine significantly increased *T. denticola* final cell density. Glycine was catabolized by *T. denticola* with approximately 73% of labeled [U-^13^C]glycine carbon being incorporated into acetate and the majority of the remainder being incorporated into lactate, suggesting that the majority of the glycine was reduced by the glycine reductase system, where ATP is generated via substrate level phosphorylation [52], [53]. The mechanism by which glycine is catabolized to lactate is less well defined but could involve conversion to serine and pyruvate, with production of NAD (Figure 8). Hence, the preferential use of exogenously acquired glycine for catabolism rather than protein biosynthesis is consistent with glycine catabolism having a major role in energy production in *T. denticola*.

*P. gingivalis* relies mainly on the uptake of peptides as a source of amino acids for energy production and it transports few free amino acids [48], [49]. In mono-culture, *P. gingivalis* did not utilize free glycine and the concentration of free glycine in the culture supernatant increased slightly over time. The addition of cell-free *T. denticola* conditioned medium to a *P. gingivalis* culture significantly increased the concentration of free glycine in the culture medium. This stimulation of free glycine production is consistent with the increased expression of *T. denticola* genes encoding glycine catabolic pathways and might partially explain the increase in *T. denticola* cell numbers during co-culture. The increase in free glycine was dependent on the presence of *P. gingivalis* and a result of an increase in the hydrolysis of glycine-containing peptides in the medium not *de novo* synthesis of glycine by *P. gingivalis*. The transcriptomic data of this current study indicated that two *P. gingivalis* genes (*PG0753* and *PG0383*) encoding putative proteases were significantly up-regulated during co-culture with *T. denticola*. It is possible that these up-regulated enzymes acting in concert are involved in the observed increase in free glycine. Whatever the precise mechanism this result represents the first demonstration of the stimulation of peptide hydrolysis by *P. gingivalis* to release free glycine in response to the presence of *T. denticola* conditioned culture fluid. During infection of a host, *P. gingivalis* glycine production would be beneficial for establishment and growth of *T. denticola*, especially as the bacterium is motile and may respond chemotactically to the amino acid.

Transcriptome analysis of *T. denticola* gene expression as a result of co-culture with *P. gingivalis* identified the up-regulation of genes encoding several known *T. denticola* virulence factors including dentilisin protease complex, major sheath protein and cystalysin, which may in part explain the observed synergistic pathogenicity of *T. denticola* and *P. gingivalis* co-infections in animal models [16], [18], [19], [54]–[58]. As some of these proteins have also been shown to be involved in coaggregation of *T. denticola* with *P. gingivalis* [59]–[62] the up-regulation of these genes might enhance the coaggregation we observed in co-culture and aid site colonisation and synergistic biofilm formation by these species.

Collectively the results of this study indicate that *P. gingivalis* and *T. denticola* sense and respond to each other's presence and exhibit metabolic symbioses during co-culture which may contribute towards their establishment and persistence in the periodontal pocket. The co-aggregation, metabolite cross-feeding and up-regulation of *T. denticola* genes encoding virulence factors help explain the temporal and spatial co-localization of the two species as surface microcolonies in subgingival plaque closely associated with chronic periodontitis [6], [12] and the synergistic virulence of these bacteria in animal models of disease [18].

## Supporting Information

Figure S1 **Correlation between microarray and qRT-PCR expression ratios.** *T. denticola* mono-culture versus co-culture gene expression ratios obtained using microarray or qRT-PCR were plotted and the correlation of coefficient determined by linear regression line (R^2^ = 0.9839).
(TIF)

Figure S2 **NMR spectra of the metabolic end products of glycine metabolism by** ***T. denticola*** **.** The metabolic end products of glycine metabolism by *T. denticola* were determined by following the fate of [U-^13^C]glycine (5 mM) added to a 24 h batch-grown *T. denticola* culture. Samples were collected every 24 h, filtered and the identity of the isotopically-labeled carbon-containing compounds were identified using NMR spectroscopy. Acetate and lactate were the major end products of *T. denticola* glycine metabolism. [^13^C_1_]acetate (184 ppm, a), [^13^C_2_]acetate (26 ppm, b), [^13^C_2_]lactate (71 ppm, c) and [^13^C_3_]acetate (23 ppm, d).
(TIF)

Protocol S1 **Gas Chromatography – mass spectrometry (GC-MS).**
(DOC)

Table S1 **Sequence of primers used in quantitative reverse transcription PCR.**
(DOC)

Table S2 ***T. denticola*** **genes differentially expressed during co-culture with** ***P. gingivalis***. Shading indicates genes predicted to be polycistronic.
(DOC)

Table S3 ***P. gingivalis*** **genes differentially expressed during co-culture with** ***T. denticola***. Shading indicates genes are predicted to be polycistronic.
(DOC)
