# Supplementary figures and images for: Porphyromonas gingivalis and Treponema denticola Exhibit Metabolic Symbioses
Source: PLoS Pathog. 2014 Mar 6;10(3):e1003955. doi: 10.1371/journal.ppat.1003955 (PMC3946380; doi:10.1371/journal.ppat.1003955)

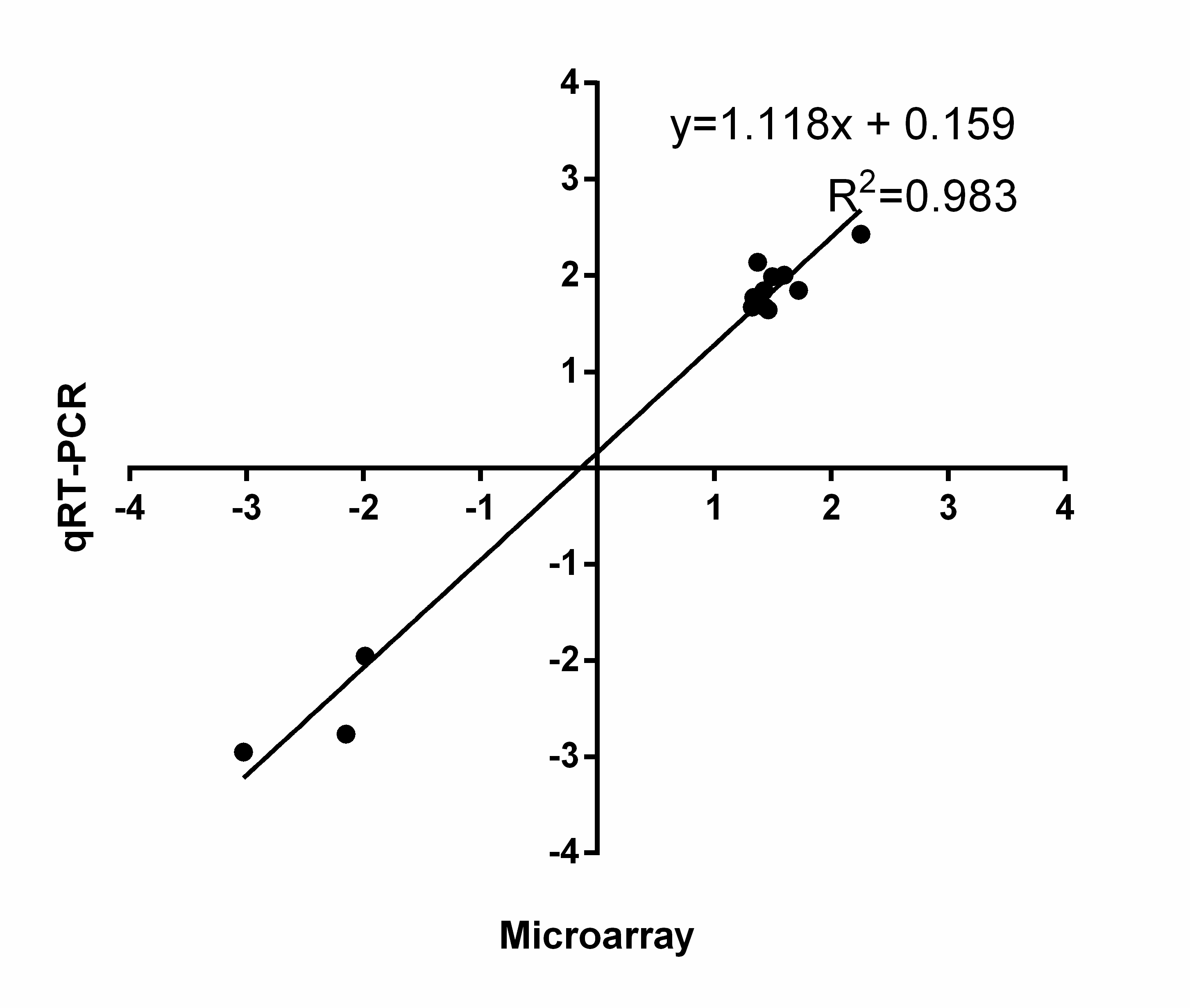

Supplement: Figure S1 — Correlation between microarray and qRT-PCR expression ratios. T. denticola mono-culture versus co-culture gene expression ratios obtained using microarray or qRT-PCR were plotted and the correlation of coefficient determined by linear regression line (R2 = 0.9839). (TIF) [file ppat.1003955.s001.tif]

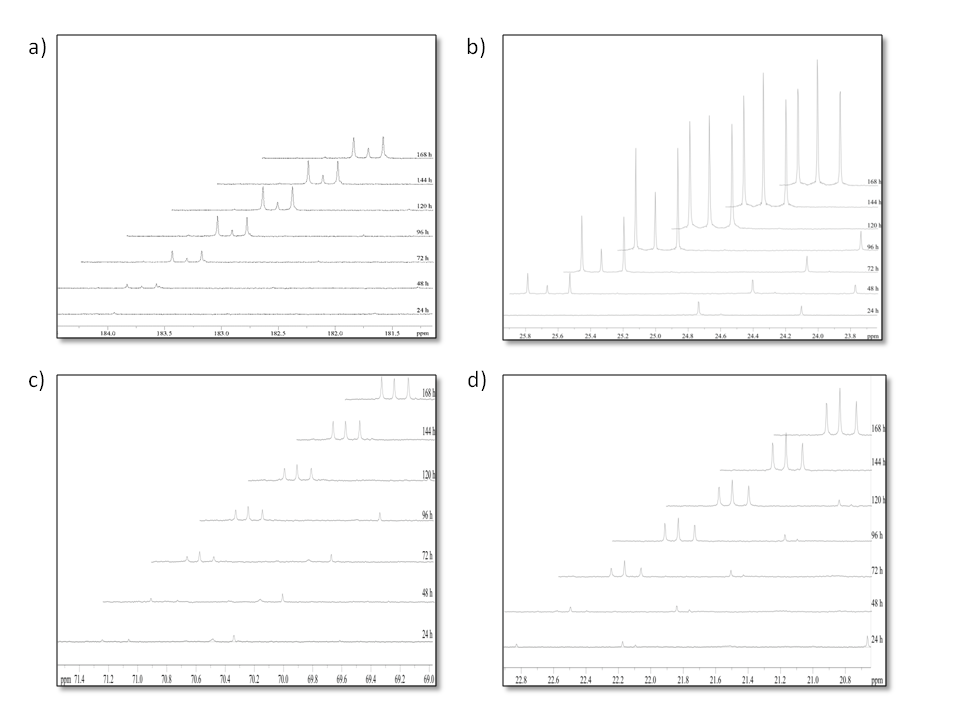

Supplement: Figure S2 — NMR spectra of the metabolic end products of glycine metabolism by T. denticola . The metabolic end products of glycine metabolism by T. denticola were determined by following the fate of [U-13C]glycine (5 mM) added to a 24 h batch-grown T. denticola culture. Samples were collected every 24 h, filtered and the identity of the isotopically-labeled carbon-containing compounds were identified using NMR spectroscopy. Acetate and lactate were the major end products of T. denticola glycine metabolism. [13C1]acetate (184 ppm, a), [13C2]acetate (26 ppm, b), [13C2]lactate (71 ppm, c) and [13C3]acetate (23 ppm, d). (TIF) [file ppat.1003955.s002.tif]
