# Supplementary material for: Porphyromonas gingivalis and Treponema denticola Exhibit Metabolic Symbioses
Source: PLoS Pathog. 2014 Mar 6;10(3):e1003955. doi: 10.1371/journal.ppat.1003955 (PMC3946380; doi:10.1371/journal.ppat.1003955)
Supplement: Protocol S1 — Gas Chromatography – mass spectrometry (GC-MS). (DOC) [file ppat.1003955.s003.doc]

**Protocol S1. Gas chromatography – mass spectrometry (GC-MS)**

Aliquots of cultures (30 µL) were collected at 0, 10, 22, 28, 36 and 46 h and diluted with 70 µL of deionized water, then further diluted with 300 µL of CHCl_3_ and 100 µL of CH_3_OH. The samples were rapidly snap-frozen on liquid nitrogen and stored at -80 °C. For GC-MS analysis, 200 µL of 50 µM scyllo-inositol in deionized water was added to the sample as an internal normalization standard. The addition of water partitioned the solution, which was improved by centrifugation at 10,000 g for 5 min at 0 °C. An aliquot of the upper aqueous phase (50 µL) was transferred into a microvial insert and dried *in vacuo* at 35 °C. Methanol (100 µL) was added to the dried extract which was subsequently dried *in vacuo* at 35 °C to remove any residual water. The dried extract was subjected to methoximation with 20 mg mL^-1^ methoxyamine in pyridine (20 µL) and incubated for at least 4 h at 30 °C with shaking. Subsequently, 20 µL of N-methyl-N-trimethylsilyl-trifuoroacetamide with 1 % v/v trimethylchlorosilane was added and then incubated for at least 1 h at 30 °C with shaking. One microliter of the treated sample was injected into a gas chromatograph (GC, Agilent Technologies 6890N Network GC System) fitted with a DB5-MS+DG column (Agilent Technologies, 30 m x 0.25 mm, 0.25 µm film thickness, with 10 m DuraGuard) coupled with a mass spectrometer (Leco Pegasus III TOF, MI, USA).
