# Supplementary material for: Porphyromonas gingivalis and Treponema denticola Exhibit Metabolic Symbioses
Source: PLoS Pathog. 2014 Mar 6;10(3):e1003955. doi: 10.1371/journal.ppat.1003955 (PMC3946380; doi:10.1371/journal.ppat.1003955)
Supplement: Table S1 — Sequence of primers used in quantitative reverse transcription PCR. (DOC) [file ppat.1003955.s004.doc]

**Table S1.** Sequence of primers used in quantitative reverse transcription PCR.

| Gene | Forward sequence ( 5’ -> 3’) | Reverse sequence ( 5’ -> 3’) |
| --- | --- | --- |
| *TDE0002* | GGACAATGGCCGAGGTATTC | AACGCCTACTCCGTGCAAAC |
| *TDE0387* | GATATCGGTTCAACGGCAAC | AAGGAACGCTCACTCTTCCA |
| *TDE0405* | CTTTTGGATGCAAAGGTCGT | ATGTAACCGCCGAAGAAATG |
| *TDE0627* | TGAGTCTGCGGTGAAAGATG | AGCCTTGCGCAAATACTGAT |
| *TDE0762* | GGCTCCGAATCAAAACGATA | CTATCGACTCCCCGTTTTCA |
| *TDE0832* | TCTGCCTGATTGGGTAAAGG | GGTTCCAGCCTAAGGAAAGG |
| *TDE0872* | AAAAGAAGCCCAGGAAAAGG | GGAAGGCCTACAACTTCAGC |
| *TDE1208* | AATGCTTCTTGCCCGAAATG | TTGCTTCTGTCCACCTCCAC |
| *TDE1226* | TTTAGTTACGGCCCATGATGC | TGCTCCTGCTTCGCTTACAG |
| *TDE1259* | GCAGGTATGGCAACCAACTT | TTACCCATGAATCCGAGAGC |
| *TDE1624* | GTGCTCACGGAGAGTTCACA | AAGCATGAGGGCAGCAGTAT |
| *TDE1625* | CCGCTGAGGAAACAAAAGAG | GTTATAAGCACCGGCACCTC |
| *TDE1626* | CAAAGCGAAATGGGAGATGT | CCGAAGGCATCTTCATTGAT |
| *TDE1627* | AGTTCGGCGGATATGAAATG | ATCTACGATTCCGCCCTTTT |
| *TDE1669* | AGTGTTCAATGCCGTTAGGG | ACCCTGCCTACAGGATTGTG |
| *TDE1999* | GGCCAAGGCAGAAAGAGTTG | TGATTTACCTTGCCCGCTTC |
| *TDE2119* | ATCCCCGCTATTTCTGGTCT | CGTACGCCTCTGGGTAAGAG |
| *TDE2120* | TATGGACGGTGCAATCGTAA | GAGCCAACTTTGCTGAAAGG |
| *TDE2535* | CCCACCAGAGCCGAAGTTAC | CGCAATTGAGGTCATCATGG |
