# Supplementary material for: Porphyromonas gingivalis and Treponema denticola Exhibit Metabolic Symbioses
Source: PLoS Pathog. 2014 Mar 6;10(3):e1003955. doi: 10.1371/journal.ppat.1003955 (PMC3946380; doi:10.1371/journal.ppat.1003955)
Supplement: Table S2 — T. denticola genes differentially expressed during co-culture with P. gingivalis. Shading indicates genes predicted to be polycistronic. (DOC) [file ppat.1003955.s005.doc]

**Table S2. *T. denticola* genes differentially expressed during co-culture with *P. gingivalis*. Shading indicates genes predicted to be polycistronic.**

| Gene Name | Fold Change (Co/Mono) | *p* value | Gene  Product | Gene Symbol | COG Category |
| --- | --- | --- | --- | --- | --- |
| *TDE0013* | 1.5 | 0 | methylenetetrahydrofolate dehydrogenase/ methenyltetrahydrofolate cyclohydrolase | *folD* | H |
| *TDE0014* | -1.9 | 0 | conserved hypothetical protein |  | unassigned |
| *TDE0015* | -1.8 | 0 | lipoprotein, putative |  | unassigned |
|  |  |  |  |  |  |
| *TDE0028* | -2.5 | 0 | ABC transporter, ATP-binding protein, HlyB family |  | V |
| *TDE0029* | -1.7 | 0 | ABC transporter, ATP-binding protein, HlyB family |  | V |
| *TDE0062* | -1.6 | 0 | PTS system, IIA component |  | T |
| *TDE0070* | -2.3 | 0 | RNA polymerase sigma-70 factor, region 2 family |  | K |
| *TDE0091* | -1.4 | 0 | RNA polymerase sigma-24 factor, putative |  | K |
| *TDE0115* | -2.9 | 0 | conserved hypothetical protein |  | R |
| *TDE0116* | -1.8 | 0.04 | membrane protein, putative |  | R |
| *TDE0117* | -1.4 | 0.01 | lipoprotein, putative |  | unassigned |
| *TDE0119* | -1.5 | 0.01 | flagellar protein FliS | *fliS* | U |
| *TDE0123* | -1.5 | 0 | conserved hypothetical protein |  | unassigned |
| *TDE0124* | -1.8 | 0 | rhomboid family protein |  | R |
| *TDE0141* | 1.6 | 0 | hypothetical protein |  | unassigned |
| *TDE0143* | 1.4 | 0 | thiamine ABC transporter, thiamine-binding protein |  | H |
| *TDE0151* | -1.5 | 0 | integral membrane protein, YeeE/YedE family |  | unassigned |
| *TDE0166* | 1.5 | 0.02 | hypothetical protein |  | unassigned |
| *TDE0167* | 1.6 | 0 | ABC transporter, ATP-binding protein |  | V |
| *TDE0168* | 1.5 | 0.03 | transcriptional regulator, GntR family |  | K |
| *TDE0205* | -1.9 | 0 | adenylate/guanylate cyclase catalytic domain protein |  | T |
| *TDE0207* | 1.5 | 0 | permease, GntP family |  | E |
| *TDE0265* | -1.5 | 0 | conserved hypothetical protein, authentic frameshift |  | unassigned |
| *TDE0332* | -1.4 | 0 | transcriptional regulator, TetR family |  | K |
| *TDE0335* | -1.4 | 0.01 | ribonucleoside-diphosphate reductase, beta subunit | *nrdB* | F |
| *TDE0337* | -1.5 | 0 | glucosamine-6-phosphate isomerase | *nagB* | G |
| *TDE0338* | -1.6 | 0 | methyl-accepting chemotaxis protein-like protein |  | unassigned |
| *TDE0339* | -1.4 | 0 | transcriptional regulator, TetR family |  | K |
| *TDE0361* | 1.5 | 0 | transporter, putative |  | E |
| *TDE0362* | -1.5 | 0 | bacterial Ig-like domain protein |  | unassigned |
| *TDE0387* | -2.1 | 0 | (R)-hydroxyglutaryl-CoA dehydratase activator | *hgdC* | I |
| *TDE0388* | -1.8 | 0 | hypothetical protein |  | unassigned |
| *TDE0389* | -2.3 | 0 | (R)-2-hydroxyglutaryl-CoA dehydratase, beta subunit, putative |  | E |
| *TDE0390* | -2 | 0 | conserved hypothetical protein |  | unassigned |
| *TDE0392* | -1.6 | 0 | (R)-2-hydroxyglutaryl-CoA dehydratase, beta subunit, putative |  | E |
| *TDE0405* | 2.3 | 0 | major outer sheath protein |  | unassigned |
| *TDE0457* | -1.4 | 0 | hypothetical protein |  | unassigned |
| *TDE0467* | -1.6 | 0 | hypothetical protein |  | unassigned |
| *TDE0468* | -1.8 | 0 | conserved hypothetical protein |  | unassigned |
| *TDE0471* | -1.6 | 0 | BNR domain protein |  | unassigned |
| *TDE0484* | -1.4 | 0.02 | methyl-accepting chemotaxis protein |  | T |
| *TDE0601* | -1.4 | 0 | malonyl CoA-acyl carrier protein transacylase, putative |  | I |
| *TDE0602* | -1.4 | 0 | 3-oxoacyl-(acyl-carrier-protein) synthase III | *fabH* | I |
| *TDE0626* | -1.6 | 0.01 | hypothetical protein |  | unassigned |
| *TDE0627* | -2 | 0 | co-chaperone protein GrpE | *grpE* | O |
| *TDE0662* | -1.4 | 0 | conserved hypothetical protein |  | S |
| *TDE0663* | -1.4 | 0.02 | conserved hypothetical protein |  | L |
|  |  |  |  |  |  |
| *TDE0666* | -1.6 | 0.04 | FeS assembly ATPase SufC | *sufC* | O |
| *TDE0667* | -1.4 | 0.03 | FeS assembly protein SufB | *sufB* | O |
|  |  |  |  |  |  |
| *TDE0677* | -1.5 | 0 | conserved hypothetical protein |  | unassigned |
| *TDE0678* | -1.4 | 0.02 | hypothetical protein |  | unassigned |
| *TDE0700* | -1.4 | 0.02 | hypothetical protein |  | unassigned |
| *TDE0709* | -1.7 | 0 | methionine-R-sulfoxide reductase | *msrA* | O |
| *TDE0718* | 1.4 | 0.02 | hypothetical protein |  | unassigned |
| *TDE0761* | 1.7 | 0 | protease complex-associated polypeptide | *prcA* | unassigned |
| *TDE0762* | 1.6 | 0 | serine protease, dentilisin |  | unassigned |
|  |  |  |  |  |  |
| *TDE0815* | 1.5 | 0.02 | membrane protein, putative |  | S |
| *TDE0816* | 1.4 | 0.05 | peptidase, M20/M25/M40 family |  | R |
|  |  |  |  |  |  |
| *TDE0832* | -3 | 0 | hypothetical protein |  | unassigned |
| *TDE0833* | -2.6 | 0 | lipoprotein, putative |  | S |
| *TDE0839* | -1.4 | 0 | rhodanese-like domain protein |  | P |
| *TDE0937* | -1.9 | 0 | RNA polymerase sigma-70 factor family protein |  | K |
| *TDE0978* | 1.5 | 0 | conserved domain protein |  | unassigned |
| *TDE0985* | 1.4 | 0.03 | oligopeptide/dipeptide ABC transporter,  periplasmic peptide-binding protein, putative |  | E |
| *TDE0994* | -1.4 | 0 | hypothetical protein |  | unassigned |
| *TDE1009* | -2.1 | 0 | methyl-accepting chemotaxis protein |  | T |
| *TDE1020* | -1.7 | 0 | dicarboxylate transporter,  periplasmic dicarboxylate-binding protein, putative |  | G |
| *TDE1035* | 1.9 | 0 | transcriptional regulator, PadR family |  | K |
| *TDE1036* | 1.7 | 0.01 | membrane protein, putative |  | unassigned |
|  |  |  |  |  |  |
| *TDE1066* | 1.4 | 0.03 | hypothetical protein |  | unassigned |
| *TDE1067* | 1.4 | 0.01 | oligopeptide/dipeptide ABC transporter, ATP-binding protein |  | E |
| *TDE1069* | 1.4 | 0.01 | oligopeptide/dipeptide ABC transporter, permease protein |  | P |
| *TDE1071* | 1.7 | 0.01 | peptide ABC transporter, peptide-binding protein OppA | *oppA* | E |
| *TDE1072* | 1.6 | 0 | lipoprotein, putative |  | R |
| *TDE1103* | -1.6 | 0 | hypothetical protein |  | unassigned |
| *TDE1188* | 1.6 | 0 | NAD(P) transhydrogenase, beta subunit | *pntB* | C |
| *TDE1189* | 1.5 | 0 | NAD(P) transhydrogenase,  alpha subunit, authentic frameshift |  | unassigned |
| *TDE1192* | -1.4 | 0.02 | hypothetical protein |  | unassigned |
| *TDE1231* | -1.9 | 0 | hypothetical protein |  | unassigned |
| *TDE1232* | -1.7 | 0 | conserved domain protein |  | S |
| *TDE1259* | 1.4 | 0.01 | amino acid carrier family protein |  | E |
| *TDE1296* | -1.5 | 0.03 | ribosomal subunit interface protein, putative |  | unassigned |
| *TDE1343* | -1.8 | 0 | trypsin domain/PDZ domain protein |  | O |
| *TDE1354* | -1.5 | 0 | hypothetical protein |  | unassigned |
| *TDE1401* | 1.4 | 0 | DedA family protein |  | S |
| *TDE1409* | 1.4 | 0.02 | flagellar filament outer layer protein FlaA, putative |  | unassigned |
| *TDE1413* | -1.4 | 0 | cytidylyltransferase/ phosphoenolpyruvate phosphomutase, putative |  | G |
| *TDE1414* | -1.4 | 0 | phosphonopyruvate decarboxylase, putative |  | R |
| *TDE1415* | -1.5 | 0 | nucleotidyl transferase/aminotransferase, class V |  | E |
| *TDE1416* | -1.4 | 0 | ABC transporter, permease protein |  | M |
| *TDE1419* | -1.4 | 0 | glycosyl transferase, group 2 family protein |  | unassigned |
| *TDE1420* | -1.6 | 0 | hypothetical protein |  | unassigned |
|  |  |  |  |  |  |
| *TDE1421* | -1.6 | 0 | hypothetical protein |  | unassigned |
| *TDE1425* | -1.4 | 0 | conserved hypothetical protein |  | unassigned |
| *TDE1426* | -1.6 | 0 | aminotransferase, DegT/DnrJ/EryC1/StrS family |  | M |
|  |  |  |  |  |  |
| *TDE1437* | -1.4 | 0 | capsular polysaccharide biosynthesis protein |  | M |
| *TDE1438* | -1.5 | 0 | glycosyl transferase, group 1 family protein |  | M |
| *TDE1441* | -1.4 | 0 | dTDP-glucose 4,6-dehydratase | *rfbB* | M |
| *TDE1525* | 1.4 | 0.01 | hypothetical protein |  | unassigned |
| *TDE1584* | -1.8 | 0 | lipoprotein, putative |  | S |
| *TDE1585* | -1.4 | 0.01 | conserved hypothetical protein |  | unassigned |
| *TDE1586* | -1.4 | 0 | conserved hypothetical protein |  | unassigned |
|  |  |  |  |  |  |
| *TDE1618* | -1.7 | 0 | conserved hypothetical protein |  | unassigned |
| *TDE1619* | -1.5 | 0.02 | conserved hypothetical protein |  | unassigned |
| *TDE1622* | 1.7 | 0 | conserved domain protein |  | unassigned |
| *TDE1624* | 1.7 | 0 | glycine cleavage system P protein, subunit 2 | *gcvP2* | E |
| *TDE1625* | 1.5 | 0 | glycine cleavage system P protein, subunit 1 | *gcvP1* | E |
| *TDE1626* | 1.4 | 0 | glycine cleavage system H protein | *gcvH* | E |
| *TDE1628* | 1.4 | 0 | hypothetical protein |  | unassigned |
| *TDE1642* | -1.9 | 0 | conserved hypothetical protein |  | unassigned |
| *TDE1648* | -1.4 | 0 | conserved hypothetical protein |  | J |
| *TDE1661* | -1.4 | 0.02 | conserved hypothetical protein |  | S |
| *TDE1669* | 1.5 | 0.04 | hemolysin |  | E |
| *TDE1724* | -1.4 | 0.01 | hypothetical protein |  | unassigned |
| *TDE1732* | -1.5 | 0.01 | conserved hypothetical protein |  | unassigned |
| *TDE1737* | 2 | 0 | hypothetical protein |  | unassigned |
| *TDE1851* | 1.4 | 0.02 | ABC transporter, permease protein, putative |  | unassigned |
| *TDE1914* | 1.9 | 0 | Holliday junction DNA helicase RuvB | *ruvB* | L |
| *TDE1925* | -1.4 | 0 | peptidyl-prolyl cis-trans isomerase, FKBP-type |  | O |
| *TDE1947* | 1.4 | 0.03 | ABC transporter, permease protein |  | R |
| *TDE1950* | 1.5 | 0 | membrane lipoprotein TmpC, putative |  | R |
| *TDE1955* | 1.4 | 0 | hypothetical protein |  | unassigned |
| *TDE1957* | 1.4 | 0.01 | hypothetical protein |  | unassigned |
| *TDE1993* | 1.4 | 0 | aspartate ammonia-lyase, putative |  | E |
| *TDE2028* | -1.4 | 0 | OmpA family protein |  | M |
| *TDE2071* | -1.5 | 0 | hypothetical protein |  | unassigned |
| *TDE2072* | -1.6 | 0 | conserved hypothetical protein |  | unassigned |
| *TDE2085* | -2.4 | 0 | amino acid kinase family protein |  | F |
| *TDE2086* | -1.9 | 0.01 | DnaJ domain protein |  | unassigned |
| *TDE2088* | -1.6 | 0 | C4 zinc finger domain protein, DksA/TraR family |  | T |
| *TDE2119* | 1.4 | 0 | glycine reductase complex selenoprotein GrdB2 | *grdB-2* | unassigned |
| *TDE2123* | -2.3 | 0.02 | ATP-dependent Clp protease adaptor protein ClpS |  | S |
| *TDE2147* | -2 | 0 | lipoprotein, putative |  | unassigned |
| *TDE2163* | -1.6 | 0 | conserved hypothetical protein |  | R |
| *TDE2164* | -1.8 | 0 | hypothetical protein |  | unassigned |
|  |  |  |  |  |  |
| *TDE2187* | 1.6 | 0 | ParB-like nuclease domain protein |  | unassigned |
| *TDE2188* | 1.8 | 0 | hypothetical protein |  | unassigned |
| *TDE2219* | -1.5 | 0 | membrane protein, putative |  | U |
| *TDE2226* | -1.7 | 0 | ABC transporter, substrate-binding protein, putative |  | P |
| *TDE2227* | -1.4 | 0 | membrane protein, putative |  | S |
| *TDE2250* | -1.4 | 0.05 | membrane protein, putative |  | S |
| *TDE2270* | -1.7 | 0 | methyl-accepting chemotaxis protein |  | T |
| *TDE2285* | -1.7 | 0 | conserved hypothetical protein |  | unassigned |
| *TDE2287* | -1.4 | 0.01 | peptidyl-prolyl cis-trans isomerase, FKBP-type |  | O |
| *TDE2298* | -1.7 | 0 | galactokinase, putative |  | G |
| *TDE2301* | -1.4 | 0.02 | FlhB domain protein |  | S |
| *TDE2302* | -1.4 | 0.04 | HD domain protein |  | T |
| *TDE2304* | -1.4 | 0 | hypothetical protein |  | unassigned |
| *TDE2308* | -1.5 | 0 | hypothetical protein |  | unassigned |
| *TDE2317* | -1.5 | 0.02 | hemolysin III | *hlyIII* | R |
| *TDE2324* | -1.6 | 0.02 | DNA-binding response regulator |  | T |
| *TDE2333* | 1.4 | 0 | sanA protein, putative |  | S |
| *TDE2340* | -1.7 | 0 | FMN-binding domain protein |  | S |
| *TDE2341* | -1.7 | 0 | membrane-associated zinc metalloprotease, putative |  | M |
| *TDE2350* | -1.5 | 0.01 | lipoprotein, putative |  | unassigned |
| *TDE2372* | -1.7 | 0 | conserved hypothetical protein |  | unassigned |
| *TDE2374* | -1.4 | 0.01 | precorrin-8W decarboxylase, putative |  | H |
| *TDE2388* | -1.4 | 0 | ATP-dependent Clp protease, proteolytic subunit ClpP | *clpP-2* | U |
| *TDE2391* | -1.6 | 0 | peptidyl-prolyl cis-trans isomerase |  | O |
| *TDE2392* | -1.5 | 0 | hypothetical protein |  | unassigned |
| *TDE2446* | 1.4 | 0 | malate dehydrogenase |  | C |
| *TDE2465* | -2.2 | 0 | hypothetical protein |  | unassigned |
| *TDE2466* | -2.2 | 0 | conserved hypothetical protein |  | O |
| *TDE2467* | -2.6 | 0 | conserved domain protein |  | unassigned |
| *TDE2473* | -1.4 | 0.03 | ribosomal protein S21 | *rpsU* | unassigned |
| *TDE2476* | -1.4 | 0 | carbamate kinase | *arcC* | E |
| *TDE2495* | -1.5 | 0.01 | conserved domain protein |  | S |
| *TDE2496* | -1.4 | 0.01 | methyl-accepting chemotaxis protein |  | unassigned |
| *TDE2498* | -1.5 | 0.01 | metallo-beta-lactamase family protein |  | C |
| *TDE2508* | 1.4 | 0.05 | hypothetical protein |  | unassigned |
| *TDE2567* | -1.6 | 0 | hypothetical protein |  | unassigned |
| *TDE2585* | 1.4 | 0.01 | methyl-accepting chemotaxis domain/ unknown domain protein |  | T |
| *TDE2591* | -2.3 | 0 | rhodanese-like domain protein |  | P |
| *TDE2600* | -2.6 | 0 | conserved hypothetical protein |  | unassigned |
| *TDE2601* | -1.8 | 0 | surface antigen, putative |  | M |
| *TDE2602* | -1.6 | 0 | outer membrane protein, putative |  | M |
| *TDE2626* | 1.4 | 0.04 | ABC transporter, permease/ATP-binding protein |  | V |
| *TDE2648* | 1.9 | 0.03 | hypothetical protein |  | unassigned |
| *TDE2649* | 2.9 | 0 | ABC transporter, ATP-binding protein |  | V |
| *TDE2650* | 2.9 | 0 | transcriptional regulator, putative |  | K |
| *TDE2673* | -1.4 | 0 | hypothetical protein |  | unassigned |
| *TDE2705* | -1.5 | 0 | ISTde3, transposase |  | unassigned |
| *TDE2708* | -1.6 | 0 | hypothetical protein |  | unassigned |
| *TDE2709* | -1.9 | 0 | BNR domain protein |  | unassigned |
| *TDE2727* | 1.4 | 0.04 | GTPase YjeQ, putative |  | R |
| *TDE2752* | 1.4 | 0.01 | conserved hypothetical protein |  | O |
