# Supplementary material for: Porphyromonas gingivalis and Treponema denticola Exhibit Metabolic Symbioses
Source: PLoS Pathog. 2014 Mar 6;10(3):e1003955. doi: 10.1371/journal.ppat.1003955 (PMC3946380; doi:10.1371/journal.ppat.1003955)
Supplement: Table S3 — P. gingivalis genes differentially expressed during co-culture with T. denticola. Shading indicates genes are predicted to be polycistronic. (DOC) [file ppat.1003955.s006.doc]

**Table S3. *P. gingivalis* genes differentially expressed during co-culture with *T. denticola*. Shading indicates genes are predicted to be polycistronic.**

| Gene  Name | Fold Change Co/Mono | *p* value | Gene  Product | Gene Symbol | COG category |
| --- | --- | --- | --- | --- | --- |
| *PG0008* | 1.9 | 0.00 | ISPg5 transposase Orf2 |  | L |
| *PG0010* | -1.5 | 0.01 | ATP-dependent Clp protease, ATP-binding subunit ClpC |  | O |
| *PG0031* | 1.7 | 0.00 | hypothetical protein |  | unassigned |
| *PG0147* | 1.4 | 0.00 | hypothetical protein |  | unassigned |
| *PG0161* | -1.4 | 0.01 | hypothetical protein |  | unassigned |
| *PG0164* | 1.4 | 0.01 | hypothetical protein |  | unassigned |
| *PG0174* | 1.7 | 0.00 | pyridine nucleotide-disulphide oxidoreductase family protein |  | R |
| *PG0275* | -1.5 | 0.00 | thioredoxin family protein |  | unassigned |
| *PG0282* | 1.4 | 0.00 | ABC transporter, ATP-binding protein |  | V |
| *PG0285* | 1.4 | 0.01 | hypothetical protein |  | M |
| *PG0286* | -1.6 | 0.00 | hypothetical protein |  | unassigned |
| *PG0300* | 1.5 | 0.00 | TPR domain protein |  | unassigned |
| *PG0323* | -1.5 | 0.00 | hypothetical protein |  | S |
| *PG0327* | 1.5 | 0.00 | hypothetical protein |  | unassigned |
| *PG0365* | 1.5 | 0.00 | 3'-5' exonuclease domain protein |  | unassigned |
| *PG0384* | 1.5 | 0.00 | MutS2 family protein |  | L |
| *PG0459* | 1.5 | 0.00 | ISPg5, transposase Orf1 |  | unassigned |
| *PG0487* | 1.9 | 0.00 | ISPg4, transposase |  | L |
| *PG0493* | 1.7 | 0.00 | hypothetical protein |  | unassigned |
| *PG0495* | -1.4 | 0.04 | hypothetical protein |  | unassigned |
| *PG0499* | 1.4 | 0.01 | hypothetical protein |  | unassigned |
| *PG0521* | -1.8 | 0.02 | co-chaperonin GroES | *groES* | O |
| *PG0524* | 1.7 | 0.00 | hypothetical protein |  | unassigned |
| *PG0553* | -1.4 | 0.01 | extracellular protease, putative |  | unassigned |
| *PG0554* | 1.5 | 0.00 | hypothetical protein |  | unassigned |
| *PG0590* | 1.4 | 0.00 | ISPg5, transposase Orf1 |  | unassigned |
| *PG0593* | -1.4 | 0.03 | HtrA protein | *htrA* | O |
| *PG0608* | 1.4 | 0.00 | hypothetical protein |  | unassigned |
| *PG0616* | -1.4 | 0.02 | thioredoxin, putative |  | unassigned |
| *PG0617* | 1.8 | 0.01 | hypothetical protein |  | unassigned |
| *PG0717* | 1.9 | 0.00 | lipoprotein, putative |  | unassigned |
| *PG0718* | 1.7 | 0.00 | hypothetical protein |  | unassigned |
| *PG0719* | 2.0 | 0.00 | sensor histidine kinase |  | T |
| *PG0720* | 1.9 | 0.00 | DNA-binding response regulator |  | T |
| *PG0736* | 1.4 | 0.00 | ribonuclease HII | *rnhB* | L |
| *PG0757* | 1.4 | 0.00 | hypothetical protein |  | unassigned |
| *PG0814* | 1.6 | 0.00 | hypothetical protein |  | unassigned |
| *PG0815* | 1.4 | 0.00 | hypothetical protein |  | unassigned |
| *PG0833* | 1.4 | 0.00 | hypothetical protein |  | unassigned |
| *PG0865* | 1.4 | 0.00 | ISPg2, transposase |  | L |
| *PG0870* | 1.6 | 0.01 | hypothetical protein |  | unassigned |
| *PG0873* | 1.4 | 0.02 | mobilizable transposon, tnpC protein |  | unassigned |
| *PG0874* | 1.6 | 0.00 | mobilizable transposon, int protein |  | L |
| *PG0875* | 1.4 | 0.01 | mobilizable transposon, tnpA protein |  | unassigned |
| *PG0876* | 1.6 | 0.00 | tRNA modification GTPase | *thdF* | R |
| *PG0906* | -1.5 | 0.00 | lipoprotein, putative |  | unassigned |
| *PG0926* | -1.4 | 0.03 | hypothetical protein |  | unassigned |
| *PG0928* | -1.8 | 0.00 | response regulator |  | T |
| *PG0969* | 1.5 | 0.00 | S-adenosylmethionine:tRNA ribosyltransferase-isomerase,  putative |  | J |
| *PG0982* | 1.4 | 0.00 | TPR domain protein |  | N |
| *PG0989* | -1.5 | 0.00 | 50S ribosomal protein L20 | *rplT* | J |
| *PG0990* | -1.6 | 0.00 | ribosomal protein L35 | *rpmI* | J |
| *PG1084* | -1.4 | 0.03 | thioredoxin family protein |  | O |
| *PG1085* | -1.5 | 0.01 | hypothetical protein |  | unassigned |
| *PG1112* | 2.0 | 0.00 | hypothetical protein |  | unassigned |
| *PG1116* | -1.4 | 0.02 | methylenetetrahydrofolate dehydrogenase/ methenyltetrahydrofolate cyclohydrolase | *folD* | H |
| *PG1169* | 1.4 | 0.01 | hypothetical protein |  | unassigned |
| *PG1176* | 1.4 | 0.01 | ABC transporter, ATP-binding protein, putative |  | V |
| *PG1179* | -1.9 | 0.02 | hypothetical protein |  | unassigned |
| *PG1208* | -1.4 | 0.02 | molecular chaperone DnaK | *dnaK* | O |
| *PG1229* | 1.6 | 0.00 | hypothetical protein |  | unassigned |
| *PG1236* | -2.0 | 0.00 | hypothetical protein |  | unassigned |
| *PG1237* | -1.9 | 0.00 | transcriptional regulator, LuxR family |  | T |
| *PG1239* | -1.4 | 0.00 | 3-oxoacyl-(acyl-carrier-protein) reductase | *fabG* | I |
| *PG1258* | -1.4 | 0.00 | DNA-binding protein HU | *hup-2* | L |
| *PG1286* | -1.6 | 0.02 | ferritin | *ftn* | P |
| *PG1314* | -1.4 | 0.01 | chorismate synthase | *aroC* | E |
| *PG1316* | -1.4 | 0.02 | hypothetical protein |  | unassigned |
| *PG1320* | 1.4 | 0.00 | ISPg1, transposase, internal deletion |  | L |
| *PG1343* | 1.4 | 0.00 | lipoate-protein ligase B | *lipB* | H |
| *PG1374* | -1.4 | 0.05 | immunoreactive 47 kDa antigen PG97 |  | unassigned |
| *PG1375* | 1.7 | 0.00 | hypothetical protein |  | unassigned |
| *PG1381* | 1.4 | 0.00 | ABC transporter, permease protein |  | P |
| *PG1439* | 1.4 | 0.00 | hypothetical protein |  | unassigned |
| *PG1453* | 1.7 | 0.00 | integrase |  | L |
| *PG1461* | 1.9 | 0.01 | hypothetical protein |  | unassigned |
| *PG1462* | 2.3 | 0.00 | hypothetical protein |  | unassigned |
| *PG1466* | 1.5 | 0.00 | hypothetical protein |  | O |
| *PG1469* | 1.5 | 0.00 | type I restriction-modification system, M subunit, putative |  | V |
| *PG1478* | 1.5 | 0.00 | conjugative transposon protein TraK |  | unassigned |
| *PG1479* | 1.9 | 0.00 | conjugative transposon protein TraJ |  | unassigned |
| *PG1481* | 1.6 | 0.00 | conjugative transposon protein TraG |  | U |
|  |  |  |  |  |  |
| *PG1485* | 1.4 | 0.00 | conjugative transposon protein TraC |  | unassigned |
| *PG1488* | 1.5 | 0.01 | hypothetical protein |  | unassigned |
| *PG1489* | 1.4 | 0.00 | hypothetical protein |  | unassigned |
| *PG1490* | 1.4 | 0.00 | TraG family protein |  | unassigned |
| *PG1496* | 1.6 | 0.00 | hypothetical protein |  | unassigned |
| *PG1501* | -1.5 | 0.01 | transcriptional regulator, tetR family |  | K |
| *PG1503* | 1.7 | 0.00 | LytB-related protein |  | I |
| *PG1504* | 1.4 | 0.01 | NAD dependent protein |  | M |
| *PG1509* | 1.5 | 0.01 | HAD-superfamily hydrolase, subfamily IA,  variant 1 family protein |  | R |
| *PG1511* | 1.6 | 0.00 | hypothetical protein |  | unassigned |
| *PG1512* | 1.4 | 0.00 | hypothetical protein |  | unassigned |
| *PG1551* | -4.8 | 0.01 | hmuY protein | *hmuY* | unassigned |
| *PG1570* | 1.6 | 0.00 | rhodanese-like domain protein |  | P |
| *PG1571* | 3.5 | 0.00 | metallo-beta-lactamase superfamily protein |  | R |
| *PG1572* | 2.2 | 0.00 | hypothetical protein |  | R |
| *PG1574* | 1.4 | 0.03 | hypothetical protein |  | unassigned |
| *PG1579* | -1.4 | 0.01 | ATPase, MoxR family |  | R |
| *PG1594* | 1.4 | 0.00 | ComEC/Rec2-related protein |  | R |
| *PG1662* | -1.5 | 0.04 | hypothetical protein |  | unassigned |
| *PG1715* | -1.4 | 0.01 | hypothetical protein |  | unassigned |
| *PG1723* | -1.4 | 0.01 | ribosomal protein S20 | *rpsT* | J |
| *PG1729* | -1.7 | 0.00 | thiol peroxidase |  | O |
| *PG1734* | 1.6 | 0.00 | transporter, putative |  | R |
| *PG1747* | 1.4 | 0.00 | ribose 5-phosphate isomerase B, putative |  | G |
| *PG1763* | -1.5 | 0.00 | ribonuclease III | *rnc* | K |
| *PG1764* | -1.6 | 0.00 | 3-oxoacyl-(acyl-carrier-protein) synthase II | *fabF* | I |
| *PG1765* | -1.7 | 0.01 | acyl carrier protein | *acpP* | I |
| *PG1769* | 1.6 | 0.00 | hypothetical protein |  | unassigned |
| *PG1784* | 1.7 | 0.00 | hypothetical protein |  | unassigned |
| *PG1788* | -1.4 | 0.05 | cysteine peptidase, putative |  | E |
| *PG1798* | -1.4 | 0.00 | immunoreactive 46 kDa antigen PG99 |  | unassigned |
| *PG1847* | 1.4 | 0.05 | endoribonuclease L-PSP, putative |  | J |
| *PG1858* | -1.7 | 0.04 | flavodoxin |  | C |
| *PG1864* | 1.6 | 0.00 | leucine-rich protein |  | S |
| *PG1898* | 1.5 | 0.03 | putative thiamine transporter, PnuT | *pnut* | H |
| *PG1910* | -1.4 | 0.00 | ribosomal protein L17 | *rplQ* | J |
| *PG2006* | -1.8 | 0.00 | hypothetical protein |  | unassigned |
| *PG2032* | 1.5 | 0.00 | primosomal protein N' | *priA* | L |
| *PG2037* | 1.7 | 0.00 | hypothetical protein |  | unassigned |
| *PG2038* | 1.5 | 0.00 | N-acetylmuramoyl-L-alanine amidase, putative |  | unassigned |
| *PG2074* | -1.5 | 0.00 | hypothetical protein |  | unassigned |
| *PG2100* | 2.0 | 0.00 | immunoreactive 63 kDa antigen PG102 |  | unassigned |
| *PG2107* | 1.8 | 0.00 | thiamine biosynthesis protein ThiH | *thiH* | H |
| *PG2108* | 1.7 | 0.00 | thiazole synthase | *thiG* | H |
| *PG2109* | 1.7 | 0.00 | thiamine-phosphate pyrophosphorylase | *thi E/D* | H |
| *PG2111* | 1.5 | 0.00 | thiS protein | *thiS* | H |
| *PG2112* | 1.9 | 0.00 | hypothetical protein |  | unassigned |
| *PG2130* | 1.7 | 0.00 | hypothetical protein |  | unassigned |
| *PG2131* | 1.7 | 0.00 | 60 kDa Fimbriae |  | unassigned |
|  |  |  |  |  |  |
| *PG2133* | 1.6 | 0.00 | lipoprotein, putative |  | unassigned |
| *PG2134* | 1.5 | 0.00 | lipoprotein, putative |  | unassigned |
| *PG2148* | 1.4 | 0.00 | xanthine/uracil permease family protein |  | F |
| *PG2213* | -1.7 | 0.01 | nitrite reductase-related protein |  | unassigned |
